# *CACUL1* promotes hepatocellular carcinoma progression through enhanced tumor cell proliferation and macrophage-mediated immune suppression

**DOI:** 10.3389/fimmu.2026.1819873

**Published:** 2026-07-16

**Authors:** Xing Wang, Yuhan Tan, Ju Wang, Ying Kong

**Affiliations:** 1 Department of Medical Oncology, The First Affiliated Hospital of Xi’an Jiaotong University, Xi’an, Shaanxi, China; 2 Department of Radiation Oncology, The Second Affiliated Hospital of Xi’an Jiaotong University, Xi’an, Shaanxi, China

**Keywords:** *CACUL1*, cell proliferation, hepatocellular carcinoma, macrophage, PI3K/AKT

## Abstract

**Background:**

Although cullin-family E3 ubiquitin ligases regulate diverse oncogenic pathways, the role of *CACUL1* in hepatocellular carcinoma (HCC) remains poorly characterized.

**Methods:**

We performed pan-cancer expression and survival analysis using TCGA and GEO databases, validated findings through immunohistochemistry, and conducted functional studies in HCC cell lines using CRISPR-mediated *CACUL1* knockout. Transcriptome sequencing, functional assays (CCK-8, wound-healing, transwell invasion), and single-cell RNA-sequencing were performed to elucidate mechanisms.

**Results:**

Pan-cancer analysis identified *CACUL1* overexpression in HCC and gastric cancer (*P* < 0.001), with prognostic significance in 16 tumor types, including HCC (log-rank *P* < 0.01). *CACUL1* expression independently predicted poor HCC prognosis (multivariable Cox, *P* < 0.05) and correlated with advanced clinicopathological features and reduced immune checkpoint expression. *CACUL1* knockout inhibited HCC cell proliferation and migration but not invasion, with transcriptomic analysis revealing PI3K/AKT signaling pathway enrichment. Co-culture experiments demonstrated that *CACUL1* promotes M2 macrophage polarization through Notch1 suppression, with pharmacological Notch inhibition phenocopying this effect.

**Conclusion:**

*CACUL1* functions as a dual-axis oncogenic driver in HCC through enhanced tumor cell proliferation associated with PI3K/AKT signaling and immune evasion via Notch1-dependent macrophage polarization, positioning it as a candidate prognostic biomarker and therapeutic target.

## Introduction

The cullin-RING E3 ubiquitin ligase (CRL) family constitutes the largest known superfamily of multi-subunit E3 ligases, with more than 300 distinct complexes identified in eukaryotes (1). CRLs function as critical molecular scaffolds that facilitate ubiquitin-dependent proteasomal degradation of diverse substrate proteins through a modular architecture: a cullin protein serves as the central scaffold that simultaneously recruits a substrate-recognition module and a catalytic RING finger domain, thereby directing the transfer of ubiquitin from E2 conjugating enzymes to target proteins. Through selective degradation of regulatory proteins, CRLs regulate numerous fundamental cellular processes including cell cycle progression, cell death, DNA repair, and stress responses, which are frequently dysregulated in human cancers (2–4). The pathogenic roles of CRL family members have been increasingly documented in tumorigenesis and tumor progression across multiple cancer types. Aberrant CRL expression and activity drive cancer development through altered substrate degradation patterns that perturb cellular homeostasis. For example, CUL4B-RING E3 ligase is frequently overexpressed in hepatocellular carcinoma (HCC) tissues and correlates with poor patient prognosis; recent evidence demonstrates that CUL4B promotes HCC cell proliferation and chemoresistance by facilitating the degradation of the RNA-binding protein FUS, thereby impairing miR-143-3p biogenesis and activating oncogenic KRAS signaling (5). Similarly, CUL2-RING ligase targets the tumor suppressor RhoB for degradation in liver cancer, and overactivation of this neddylation-CRL pathway promotes hepatocarcinogenesis while suppression of this pathway restores RhoB-mediated apoptosis (6). Additionally, CUL4A-mediated ubiquitination of LATS1 has been implicated in suppressing the Hippo tumor-suppressor pathway in HCC. These findings collectively establish the CRL family as critical oncogenic drivers in hepatocarcinogenesis. While individual cullin family members (CUL1, CUL3, CUL4A/B, and CUL7) have been extensively studied in various malignancies, *CACUL1* (CDK2-associated cullin domain 1), a key structural member of the cullin family, has received limited investigation in the context of hepatocellular carcinoma despite emerging evidence of its oncogenic potential in other cancer types.

*CACUL1*, also known as CAC1, is a cullin-domain–containing protein within the CRL family that has been reported to interact directly with cyclin-dependent kinase 2 (CDK2) and to promote cell proliferation via a non-canonical mechanism that is independent of the conventional SCF complex architecture (7). In contrast to canonical cullins (CUL1–5), which mainly serve as scaffolds for multi-subunit ubiquitin ligase assemblies, *CACUL1* shows cell cycle–dependent expression and has been implicated as a positive regulator of cell proliferation by enhancing CDK2 kinase activity. Recent studies further suggest that *CACUL1* dysregulation is associated with malignant phenotypes across multiple tumor types, including reports that *CACUL1* overexpression promotes proliferation in gastric cancer through CDK2-related mechanisms, contributes to colorectal cancer progression by supporting chemoresistance and G1/S transition in drug-resistant cells, and enhances lung cancer tumorigenicity through mechanisms linked to miRNA-associated regulatory networks (8). Nevertheless, the mechanistic basis and pathophysiological relevance of *CACUL1* in hepatocellular carcinoma remain to be fully elucidated.

To systematically characterize the oncogenic role of CACUL1, we initially performed a pan-cancer analysis integrating gene expression profiles and prognostic data. Upon identifying significantly elevated *CACUL1* expression specifically in HCC, we validated these findings in clinical specimens using immunohistochemistry and correlated expression levels with clinicopathological features and immunotherapeutic responses. Mechanistically, we employed CRISPR/Cas9-mediated gene knockout in HCC cell lines followed by transcriptome sequencing to delineate CACUL1-associated transcriptional changes. Finally, we elucidated the impact of *CACUL1* on malignant phenotypes and the tumor immune microenvironment through comprehensive *in vitro* and *in vivo* assays.

## Methods

### Data and sample acquisition

Pan-cancer transcriptomic data were obtained from the UCSC Xena platform (https://xenabrowser.net/) to systematically investigate the relationship between *CACUL1* expression and different tumor types. RNA sequencing data in Transcripts Per Million (TPM) format along with corresponding clinical phenotype data were downloaded for 33 cancer types from The Cancer Genome Atlas (TCGA) database, encompassing both tumor samples and matched normal tissues where available. For hepatocellular carcinoma (LIHC) specifically, we retrieved data from 374 tumor samples and 50 adjacent normal liver tissues with comprehensive clinical information including survival outcomes, TNM staging, age, gender, and serological markers such as alpha-fetoprotein (AFP), creatinine, and albumin levels. The independent validation cohort GSE76427, comprising 115 HCC tumor samples and 52 adjacent normal liver tissues, was downloaded from the Gene Expression Omnibus (GEO) database using the GEOquery R package (version 2.66.0) to validate the differential expression characteristics of *CACUL1* in liver cancer. Additionally, the single-cell RNA sequencing dataset GSE146115 was obtained from the GEO database to analyze CACUL1 gene expression at the single-cell resolution and to investigate cellular heterogeneity within the tumor microenvironment. Human tissue microarrays containing 10 paired HCC tumor and adjacent normal tissue samples were collected from patients who underwent surgical resection at our institution. All tissue specimens were formalin-fixed and paraffin-embedded following standard histological procedures. Sample collection and utilization were approved by the Institutional Ethics Committee (Approval No.: SHYJS-CP-2210040), and written informed consent was obtained from all participants in accordance with the Declaration of Helsinki.

### Pan-cancer expression and prognostic analysis

Differential expression analysis across TCGA pan-cancer datasets was performed using the limma R package (version 3.54.0). Prior to analysis, TPM values were log_2_-transformed (log_2_[TPM + 1]) to achieve normal distribution and stabilize variance across the expression range. The limma-trend pipeline was employed with empirical Bayes moderation to identify differentially expressed genes between tumor and normal tissues for each cancer type. Genes meeting the criteria of *P* < 0.05 and |logFC| > log_2_(1.6) (equivalent to 1.6-fold change) were considered significantly dysregulated. Multiple testing correction was applied using the Benjamini-Hochberg false discovery rate (FDR) method. For survival analysis, the survival (version 3.5.0) and survminer (version 0.4.9) R packages were utilized to assess the prognostic significance of *CACUL1* expression across different cancer types. The optimal cutoff value for stratifying patients into high- and low-expression groups was determined using the maximally selected rank statistics method implemented in the survminer package via the surv_cutpoint() function, which identifies the cutpoint that yields the most significant log-rank test statistic. Kaplan-Meier survival curves were generated using the ggsurvplot() function, and statistical significance was evaluated by log-rank tests. Univariate and multivariate Cox proportional hazards regression analyses were performed to identify independent prognostic factors, with hazard ratios (HR) and 95% confidence intervals (CI) calculated for *CACUL1* expression after adjusting for clinical covariates including age, gender, TNM stage, and tumor grade.

### Single-cell RNA sequencing analysis

The GSE146115 single-cell RNA sequencing dataset was processed using the Seurat R package (version 4.3.0) following the standard workflow. The GSE146115 scRNA-seq dataset was generated using Fluidigm C1 mRNA-Seq HT IFC (10–17 μm) and sequenced on Illumina HiSeq platform (2 × 150 bp). Because the Fluidigm C1 platform physically captures individual cells in isolated microfluidic chambers, which are subsequently verified by imaging to exclude multi-cell wells (as reported in the original study), the inherent doublet rate is negligible; thus, additional computational doublet removal was deemed unnecessary.

Quality control was performed by filtering cells with fewer than 200 detected genes, more than 6,000 genes (to further exclude any potential doublets), or mitochondrial gene content exceeding 15%. Initial quality control filtering based on total counts, total features, and ERCC spike-in percentage retained 2,064 cells from an initial 3,200 cells using SC3 consensus clustering with 1 MAD threshold. HCC8 samples were excluded due to tumor cell size exceeding the C1 HT IFC capture range. For our rigorous re-analysis, a final total of 1,950 high-quality cells were retained. The median number of detected genes per cell was 2,145, and the median number of unique molecular identifiers (UMIs) per cell was 6,830.

Raw count matrices were normalized using the NormalizeData() function with the LogNormalize method and a scale factor of 10,000. Highly variable features were identified using the FindVariableFeatures() function with the “vst” selection method, retaining the top 2000 variable genes for downstream analysis. Data were scaled using the ScaleData() function to regress out unwanted sources of variation including mitochondrial gene percentage and cell cycle effects. To account for potential technical variations and batch effects across different patient samples, the dataset was integrated using the standard canonical correlation analysis (CCA) approach via the FindIntegrationAnchors() and IntegrateData() functions implemented in Seurat.

Principal component analysis (PCA) was performed using the RunPCA() function, and the optimal number of principal components was determined by examining the elbow plot and jackstraw analysis. Unsupervised clustering was conducted using the FindNeighbors() and FindClusters() functions with a resolution parameter of 0.5. Dimensionality reduction for visualization was achieved using t-Distributed Stochastic Neighbor Embedding (t-SNE) via the RunTSNE() function, following standard Seurat workflow.

Cell type annotation was performed based on the expression of canonical marker genes and validated using the SingleR package (version 2.0.0) with reference to the Human Primary Cell Atlas. The final annotated dataset consisted of the following subpopulations: 845 malignant hepatocytes, 320 monocytes/macrophages, 410 T cells, 215 B cells, and 160 stromal/other cells. CACUL1 expression patterns across different cell populations were visualized using the FeaturePlot() and VlnPlot() functions.

Cell-cell communication analysis was conducted using CellChat (version 1.6.0) to infer intercellular signaling networks. The createCellChat() function was used to create CellChat objects for CACUL1-high and CACUL1-low tumor cell populations. Ligand-receptor interaction databases were loaded from CellChatDB, and communication probabilities were computed using the computeCommunProb() function. Differential interaction analysis between CACUL1-high and CACUL1-low groups was performed using the rankNet() function to identify signaling pathways with significant alterations. Network centrality analysis was conducted to identify major signaling sources and targets within the tumor microenvironment.

### Immunohistochemistry

Tissue microarray sections (4 μm thickness) were deparaffinized in xylene and rehydrated through a graded ethanol series (100%, 95%, 85%, 75%, and distilled water). Antigen retrieval was performed by immersing slides in 10 mM sodium citrate buffer (pH 6.0) and heating in a pressure cooker at 95°C for 20 minutes, followed by cooling at room temperature for 30 minutes. Endogenous peroxidase activity was quenched by incubation with 3% hydrogen peroxide in methanol for 10 minutes at room temperature. Non-specific binding was blocked with 5% bovine serum albumin (BSA) in phosphate-buffered saline (PBS) for 1 hour at room temperature. Sections were then incubated overnight at 4°C in a humidified chamber with primary antibodies: anti-*CACUL1* (1:200 dilution, catalog #16139-1-AP, Proteintech, Rosemont, IL, USA) or anti-Ki-67 (1:300 dilution, catalog #ab15580, Abcam, Cambridge, UK). After washing three times with PBS-Tween 20 (PBST), sections were incubated with horseradish peroxidase (HRP)-conjugated secondary antibody using the Dako EnVision+ System-HRP (DAB) Kit (catalog #K5007, Dako, Glostrup, Denmark) for 30 minutes at room temperature. Immunoreactivity was visualized by incubating with 3,3’-diaminobenzidine (DAB) substrate solution for 3–5 minutes, and the reaction was terminated by rinsing with distilled water. Sections were counterstained with hematoxylin for 1 minute, dehydrated through graded alcohols, cleared in xylene, and mounted with neutral balsam. Staining was evaluated independently by two pathologists blinded to clinical information.

### Cell culture

Human hepatocellular carcinoma cell line HepG2 (ATCC HB-8065) and murine macrophage cell line RAW264.7 (ATCC TIB-71) were obtained from the American Type Culture Collection (ATCC, Manassas, VA, USA). HepG2 cells were cultured in Dulbecco’s Modified Eagle Medium (DMEM, catalog #11965092, Gibco, Thermo Fisher Scientific, Waltham, MA, USA) supplemented with 10% fetal bovine serum (FBS, catalog #10270106, Gibco) and 1% penicillin-streptomycin (catalog #15140122, Gibco). RAW264.7 cells were maintained in DMEM with 10% FBS and 1% penicillin-streptomycin. All cells were cultured at 37°C in a humidified incubator containing 5% CO_2_. Cells were routinely passaged every 2–3 days when reaching 80-90% confluence using 0.25% trypsin-EDTA (catalog #25200056, Gibco). Cell line authentication was performed via short tandem repeat (STR) profiling by the ATCC Authentication Service within the past 6 months. Mycoplasma contamination testing was routinely conducted every 2 months using the MycoAlert Mycoplasma Detection Kit (catalog #LT07-318, Lonza, Basel, Switzerland) to ensure cell culture integrity.

### *CACUL1* knockout

CRISPR-Cas9-mediated genome editing of the *CACUL1* gene was accomplished using CRISPR-Cas9 ribonucleoprotein (RNP) complexes (Haixing Bioscience) comprising human codon-optimized Streptococcus pyogenes Cas9 (hSpCas9) and a synthetic chimeric guide RNA. A guide RNA (gRNA) targeting exon 1 of the *CACUL1* gene (5’-TTCCTGTCCACGGAGACCGCGGG-3’) was designed and validated using the online CRISPR design tool (crispr.mit.edu) to assess potential off-target effects. The gRNA expression plasmid was delivered into exponentially growing cells via nucleofection using the Neon Transfection System (Thermo Fisher Scientific) following the manufacturer’s protocol. Specifically, cells were subjected to a single pulse: 1) with a final plasmid concentration. At 48 hours post-transfection, successfully transfected cells were isolated via single-cell dilution cloning and seeded into 96-well plates containing complete culture medium to allow clonal expansion. To screen for insertions and deletions (indels) at the *CACUL1* targeting site, genomic DNA was extracted from individual clones using the Quick-DNA Miniprep Kit (Zymo Research, Catalog#: D3025). PCR amplification was performed using 2× Taq Master Mix (Dye Plus; Vazyme, Catalog#: P112) with the following primer sets flanking exon 1: Forward primer: 5’-GATGTAACTCGGGGAGGCAG-3’; Reverse primer: 5’-TGGCTGCAGAAGACTGAAGG-3’. The PCR reaction mixture consisted of template DNA, each primer, and 2× Taq Master Mix, with thermal cycling conditions. Plasmids from 8–10 individual clones displaying aberrant PCR band patterns were prepared using a standard plasmid isolation protocol and sent for bidirectional Sanger sequencing (GENEWIZ, China) to confirm the presence and nature of *CACUL1* mutations. Clones harboring confirmed biallelic mutations in the *CACUL1* gene were selected for further investigation. A panel of cell lines was established, including: wild-type, heterozygous with variants A and B designated as *CACUL1* and CACUL1, respectively), and homozygous knockout clones. All cell lines were cultured in DMEM supplemented with 10% fetal bovine serum (FBS) at 37°C in a humidified atmosphere with 5% CO_2_.

### Western blot

Cells were lysed on ice for 30 minutes in RIPA lysis buffer (catalog #89900, Thermo Fisher Scientific) supplemented with protease inhibitor cocktail (catalog #4693159001, Roche, Basel, Switzerland) and phosphatase inhibitor cocktail (catalog #4906845001, Roche). Cell lysates were centrifuged at 12,000 × g for 15 minutes at 4°C, and the supernatants were collected. Protein concentration was determined using the Pierce BCA Protein Assay Kit (catalog #23227, Thermo Fisher Scientific) according to the manufacturer’s instructions. Equal amounts of protein (30 μg per lane) were denatured by boiling in 1× SDS-PAGE loading buffer containing β-mercaptoethanol for 5 minutes at 95°C. Proteins were separated by electrophoresis on 10% SDS-polyacrylamide gels (catalog #4561034, Bio-Rad, Hercules, CA, USA) at 80 V for 30 minutes followed by 120 V for 90 minutes. Separated proteins were transferred onto 0.45 μm polyvinylidene difluoride (PVDF) membranes (catalog #IPVH00010, Millipore, Burlington, MA, USA) using a wet transfer system at 200 mA for 2 hours at 4°C. Membranes were blocked with 5% non-fat dry milk in Tris-buffered saline containing 0.1% Tween-20 (TBST) for 1 hour at room temperature with gentle shaking. Membranes were then incubated overnight at 4°C with the following primary antibodies diluted in 5% BSA-TBST: anti-*CACUL1* (1:1000, catalog #16139-1-AP, Proteintech), anti-CD163 (1:1000, catalog #ab182422, Abcam), anti-CD206 (1:1000, catalog #18704-1-AP, Proteintech), anti-Notch1 (1:1000, catalog #3608, Cell Signaling Technology, Danvers, MA, USA), anti-TNF-α (1:1000, catalog #11948, Cell Signaling Technology), and anti-β-actin (1:5000, catalog #4970, Cell Signaling Technology). After washing three times with TBST for 10 minutes each, membranes were incubated with HRP-conjugated secondary antibodies (anti-rabbit IgG, 1:5000, catalog #7074; anti-mouse IgG, 1:5000, catalog #7076; Cell Signaling Technology) for 1 hour at room temperature. Protein bands were visualized using the Enhanced Chemiluminescence (ECL) detection system (catalog #RPN2232, GE Healthcare, Chicago, IL, USA) and imaged on a ChemiDoc MP Imaging System (Bio-Rad). Band intensities were quantified using Image Lab software (version 6.0.1, Bio-Rad), and protein expression levels were normalized to β-actin loading control.

### Cell functional assays

Cell proliferation was assessed using the Cell Counting Kit-8 (CCK-8, catalog #CK04, Dojindo Molecular Technologies, Kumamoto, Japan). HepG2 cells (wild-type or CACUL1-KD) were seeded in 96-well plates at a density of 3,000 cells per well in 100 μL complete DMEM. Five replicate wells were prepared for each condition. At 24, 48, 72, and 96 hours post-seeding, 10 μL CCK-8 solution was added to each well and incubated for 2 hours at 37°C in the dark. Absorbance was measured at 450 nm using a SpectraMax i3x Multi-Mode Microplate Reader (Molecular Devices, San Jose, CA, USA). Cell viability was calculated as:


Viability(%)=[(OD_sample-OD_blank)/(OD_control-OD_blank)]×100%

For wound healing migration assays, cells were seeded in 6-well plates at a density of 5 × 10^5^ cells per well and cultured until reaching 90-95% confluence. A sterile 200 μL pipette tip was used to create a linear scratch wound across the cell monolayer. Detached cells were removed by washing twice with PBS. Serum-free DMEM was added to eliminate the effect of proliferation on migration. Images of the wound area were captured at 0, 12, and 24 hours using an EVOS M7000 Imaging System (Thermo Fisher Scientific) at 100× magnification. Wound width was measured at five random positions per image using ImageJ software (version 1.54f, National Institutes of Health, Bethesda, MD, USA).


Migration rate(%)=[(Initial width-Final width)/Initial width]×100%

Colony formation assays were performed by seeding 500 cells per well in 6-well plates containing 2 mL complete DMEM. Cells were cultured for 14 days with medium changed every 3 days. Colonies were fixed with 4% paraformaldehyde (catalog #P6148, Sigma-Aldrich) for 15 minutes and stained with 0.5% crystal violet solution (catalog #C0775, Sigma-Aldrich) for 30 minutes at room temperature. After washing with PBS and air-drying, colonies containing more than 50 cells were counted manually under a light microscope. Images were captured using a ChemiDoc Imaging System (Bio-Rad). Colony formation rate was calculated as:


Colony formation rate(Number of colonies/Number of seeded cells)×100%

Transwell invasion assays were performed using 24-well Corning BioCoat Matrigel Invasion Chambers with 8 μm pore size (catalog #354480, Corning, Corning, NY, USA). Matrigel-coated membranes were rehydrated with serum-free DMEM for 2 hours at 37°C before use. A total of 2 × 10^4^ cells in 200 μL serum-free DMEM were seeded into the upper chamber, while 600 μL DMEM containing 20% FBS was added to the lower chamber as a chemoattractant. After 24 hours of incubation at 37°C, non-invading cells on the upper surface of the membrane were carefully removed using cotton swabs. Invaded cells on the lower surface were fixed with 4% paraformaldehyde for 15 minutes and stained with 0.5% crystal violet for 30 minutes. Five random fields per membrane were photographed under a Leica DMi8 inverted microscope (Leica Microsystems, Wetzlar, Germany) at 200× magnification, and invaded cells were counted manually. Each experiment was performed in triplicate.

### Xenograft tumor model

All animal experiments were conducted in accordance with the Guide for the Care and Use of Laboratory Animals (8th edition, National Research Council) and approved by the Institutional Animal Care and Use Committee (IACUC Approval No.: XJTUAE2023-2317). Five-week-old male BALB/c nude mice (body weight 18–20 g) were purchased from Shanghai SLAC Laboratory Animal Co., Ltd. (Shanghai, China) and housed in specific pathogen-free (SPF) facilities under a 12-hour light/dark cycle with free access to food and water. After one week of acclimatization, mice were randomly divided into two groups (n = 6 per group): wild-type HepG2 xenograft group and CACUL1-KD HepG2 xenograft group. Cells (5 × 10^6^ cells per mouse) were harvested during logarithmic growth phase, washed twice with PBS, and resuspended in 100 μL sterile PBS. Cell suspensions were subcutaneously injected into the right flank of each mouse using a 1 mL syringe with a 26-gauge needle. Tumor formation was monitored daily, and once palpable tumors appeared (approximately 7 days post-injection), tumor dimensions were measured every 3 days using digital calipers. Tumor volume was calculated using the modified ellipsoid formula:


$$\text{Volume}({\text{mm}}^{3})=\text{Length}\times {\text{Width}}^{2})/2,$$

where length represents the longest diameter and width represents the perpendicular diameter. Body weight was recorded simultaneously to monitor animal health. After 4 weeks, mice were euthanized by CO_2_ inhalation followed by cervical dislocation. Tumors were immediately excised, photographed, and weighed. Tumor tissues were divided into two portions: one portion was fixed in 10% neutral-buffered formalin for 24 hours and subsequently embedded in paraffin for histological analysis, while the other portion was snap-frozen in liquid nitrogen and stored at -80°C for protein extraction.

### Statistical analysis

All statistical analyses were performed using R software (version 4.3.0) and GraphPad Prism (version 9.0, GraphPad Software, San Diego, CA, USA). Data are presented as mean ± standard deviation (SD) for continuous variables or as frequency (percentage) for categorical variables. Normality of continuous data was assessed using the Shapiro-Wilk test. For normally distributed data, comparisons between two groups were performed using two-tailed Student’s t-test, while non-normally distributed data were analyzed using the Mann-Whitney U test (Wilcoxon rank-sum test). Comparisons among three or more groups were conducted using one-way analysis of variance (ANOVA) followed by Tukey’s *post hoc* test for normally distributed data or Kruskal-Wallis test followed by Dunn’s multiple comparison test for non-normally distributed data. Categorical variables were compared using χ² test or Fisher’s exact test when appropriate. Correlations between *CACUL1* expression and clinical parameters or other gene expression levels were evaluated using Pearson correlation coefficient for normally distributed data or Spearman rank correlation coefficient for non-normally distributed data. Survival analysis was performed using the Kaplan-Meier method with log-rank test to compare survival differences between high and low *CACUL1* expression groups. Univariate and multivariate Cox proportional hazards regression models were constructed to identify independent prognostic factors, with results presented as hazard ratios (HR) and 95% confidence intervals (CI). Unless otherwise specified, HRs are reported with the low-expression group as the reference category (i.e., Low vs. High), where HR < 1 indicates reduced survival in the high-expression group. The proportional hazards assumption was verified using Schoenfeld residuals test. Time-dependent receiver operating characteristic (ROC) curves were generated using the timeROC package to evaluate the predictive accuracy of *CACUL1* expression at 1-, 3-, and 5-year time points, with area under the curve (AUC) values calculated. For differential expression analysis, the limma package was employed with empirical Bayes moderation, and P-values were adjusted for multiple testing using the Benjamini-Hochberg false discovery rate (FDR) method. For RNA sequencing data, the edgeR package was used with quasi-likelihood F-test, and genes with fold change > 1.5 and FDR < 0.05 were considered significantly differentially expressed. Functional enrichment analysis was conducted using the clusterProfiler package with hypergeometric test, and pathways with adjusted P-value < 0.05 were considered significantly enriched. For cell-based experiments, at least three independent biological replicates were performed, and statistical significance was determined by appropriate tests as described above. A two-tailed P-value < 0.05 was considered statistically significant unless otherwise specified. Significance levels are indicated as follows: *P < 0.05, **P < 0.01, ***P < 0.001, and ****P < 0.0001.

## Results

### *CACUL1* is significantly upregulated in hepatocellular carcinoma across pan-cancer profiling

**Figure 1:**
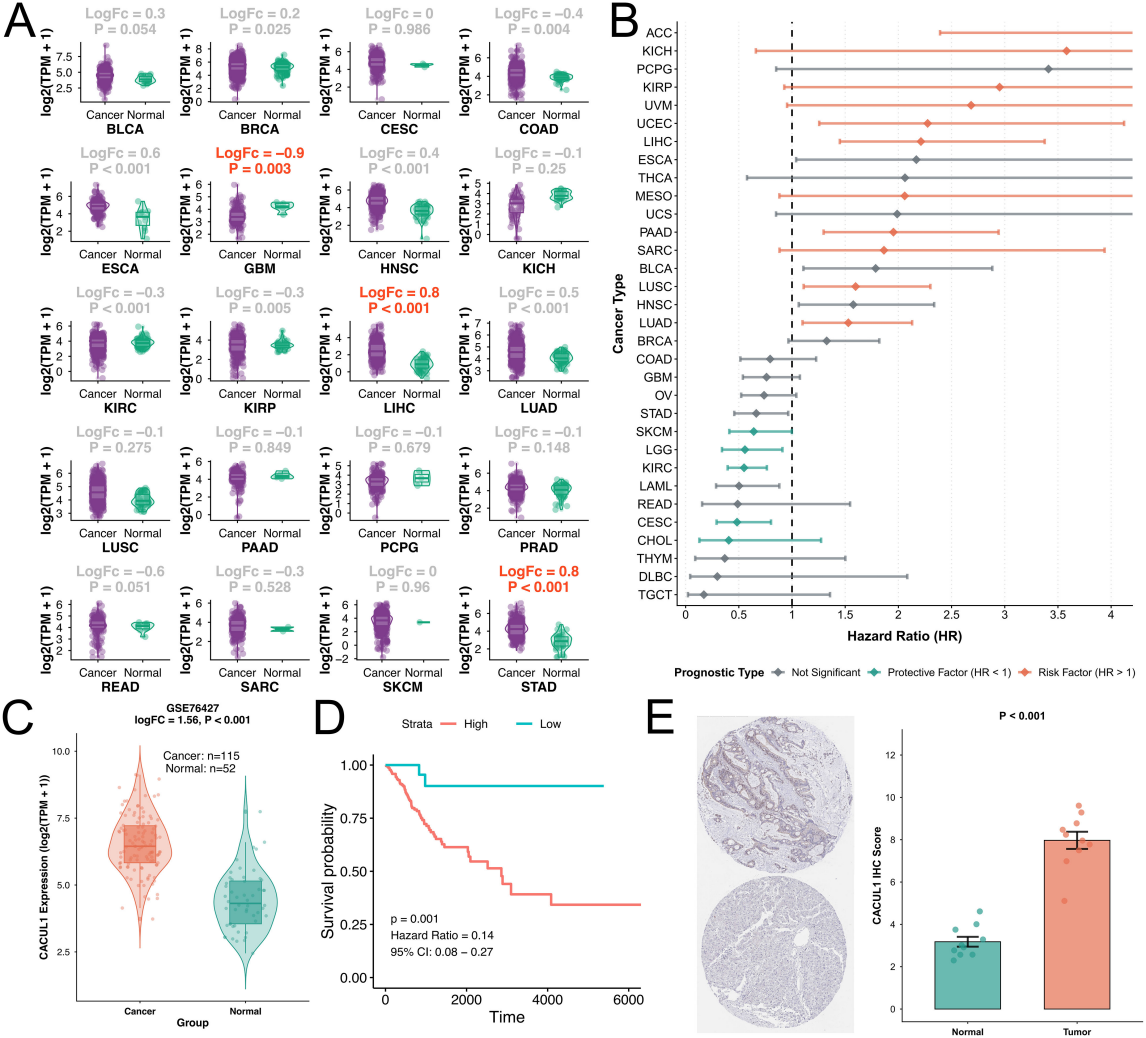
*CACUL1* is significantly upregulated in hepatocellular carcinoma across pan-cancer profiling. **(A)** Pan-cancer differential expression analysis of *CACUL1* across TCGA tumor types. Significant upregulation was observed in LIHC and STAD (*P* < 0.001), while GBM showed downregulation (*P* = 0.003). **(B)** Forest plot of univariate Cox regression showing *CACUL1* prognostic significance across 33 cancer types. Cancer types are color-coded as risk factor (HR > 1), protective factor (HR < 1), or not significant. **(C)** Validation of *CACUL1* overexpression in the GSE76427 cohort (115 tumors vs. 52 normals; LogFC = 1.56, *P* < 0.001). **(D)** Kaplan–Meier survival analysis in GSE76427 showing reduced survival in high-*CACUL1* patients (Low vs. High: HR = 0.14, 95% CI: 0.08–0.27, P = 0.001). **(E)** Representative IHC staining of *CACUL1* in paired HCC and adjacent normal tissues (n = 10 pairs; *P* < 0.001).

To systematically characterize the dysregulation of *CACUL1* across malignancies, we interrogated TCGA datasets to profile its expression landscape. While *CACUL1* expression remained stable across most tumor types, significant upregulation was observed in hepatocellular carcinoma (LIHC) and stomach adenocarcinoma (STAD) (*P* < 0.001), contrasted by downregulation in glioblastoma multiforme (GBM), suggesting tissue-specific functional roles (**Figure 1A**). We next evaluated the prognostic significance of CACUL1, revealing a broader impact on survival than on expression levels alone. *CACUL1* levels were significantly correlated with patient prognosis in 16 cancer types; specifically, high expression was associated with poor outcomes in 11 types, including LIHC and kidney renal papillary cell carcinoma (KIRP), whereas it predicted favorable survival in five types, such as kidney renal clear cell carcinoma (KIRC) and brain lower grade glioma (LGG) (**Figure 1B**). Intriguingly, *CACUL1* exhibited divergent prognostic associations within renal malignancies (KIRC vs. KIRP), underscoring the heterogeneity of its oncogenic potential. Although upregulated in STAD, *CACUL1* showed no significant prognostic correlation in this cancer type. Given the consistent upregulation and prognostic relevance in liver cancer, we focused subsequent validation on HCC. Analysis of the independent GSE76427 cohort (115 tumor vs. 52 normal) confirmed that *CACUL1* is significantly overexpressed (**Figure 1C**) and that elevated levels correlate with reduced survival (**Figure 1D**; Low vs. High: HR = 0.14, 95% CI: 0.08–0.27, P = 0.001). (**Figure 1D**). These findings were further corroborated by immunohistochemical staining of clinical specimens (n=10 pairs), which demonstrated distinct *CACUL1* protein upregulation in HCC tissues compared to adjacent normal controls (**Figure 1E**).

### *CACUL1* expression correlates with advanced clinicopathological features in hepatocellular carcinoma

**Figure 2:**
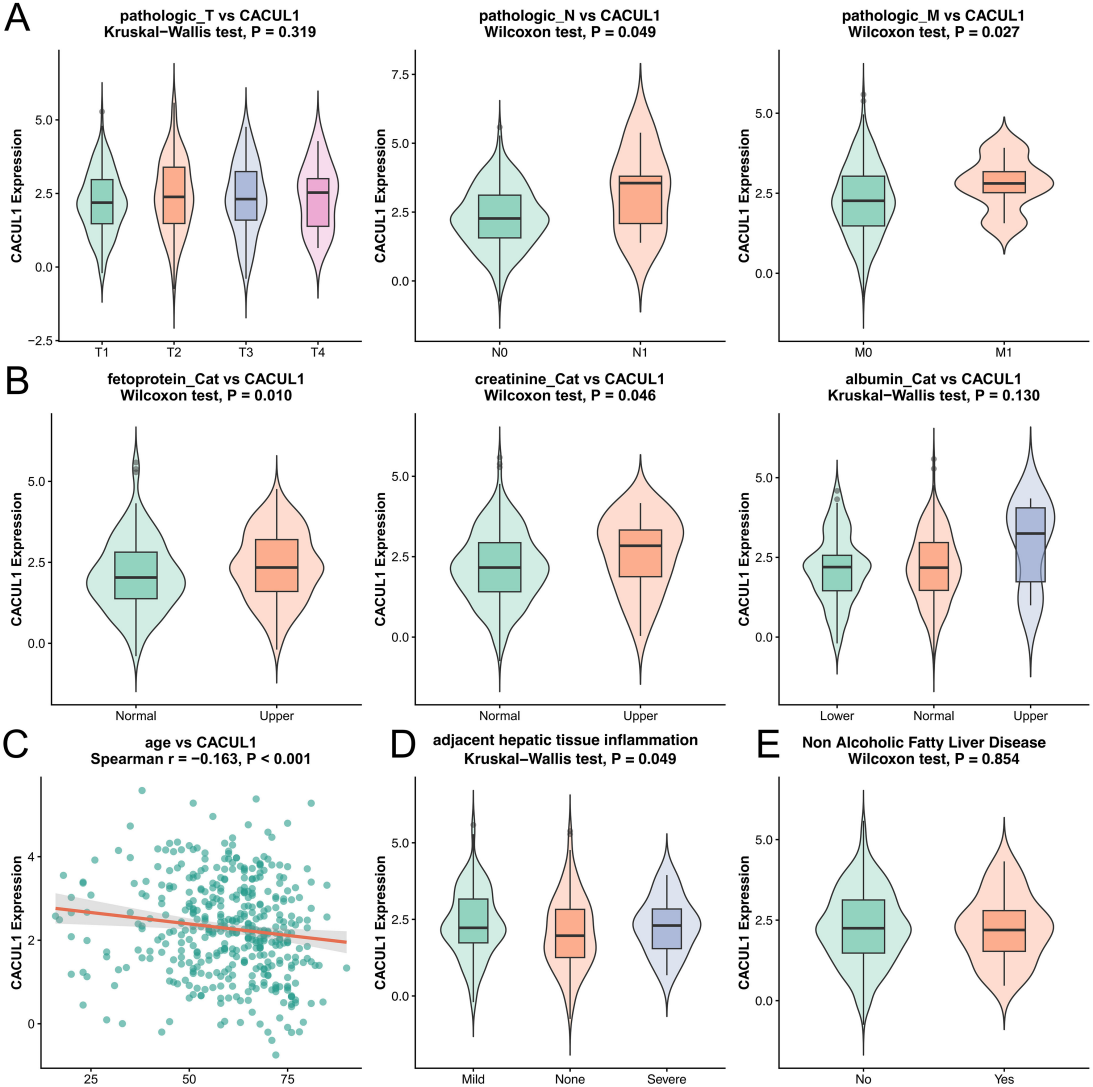
*CACUL1* expression correlates with advanced clinicopathological features in HCC. **(A)** *CACUL1* expression across TNM stages: pathologic T (*P* = 0.319), N (N0 vs. N1, *P* = 0.049), and M (M0 vs. M1, *P* = 0.027). **(B)** *CACUL1* expression stratified by AFP (*P* = 0.010), creatinine (*P* = 0.046), and albumin levels (*P* = 0.130, not significant). **(C)** Inverse correlation between *CACUL1* expression and patient age (Spearman r = −0.163, *P* < 0.001). **(D)** *CACUL1* expression across adjacent hepatic inflammation grades (*P* = 0.049). **(E)** *CACUL1* expression by NAFLD status (*P* = 0.854, not significant).

To further elucidate the clinical relevance of CACUL1, we investigated the association between its expression and various clinicopathological characteristics in HCC. Analysis of TNM staging revealed a significant correlation between elevated *CACUL1* expression and advanced disease progression. Specifically, *CACUL1* levels were markedly higher in patients with lymph node metastasis (N1 vs. N0) and distant metastasis (M1 vs. M0) (**Figure 2A**), a finding that aligns with our observation that high *CACUL1* expression predicts poor prognosis. Beyond anatomical staging, we examined correlations with serological markers; *CACUL1* expression exhibited a positive correlation with levels of alpha-fetoprotein (AFP) and creatinine (**Figure 2B**). No significant correlation was observed with albumin levels (P = 0.130). These findings suggest a potential link between CACUL1 burden and hepatic tumor load or injury. Notably, we observed a negative correlation between *CACUL1* expression and patient age (**Figure 2C**). We speculate that this inverse relationship may be attributable to the higher proliferative index and more aggressive biological behavior often observed in early-onset HCC compared to elderly cases, necessitating higher levels of cell-cycle regulators like CACUL1. Furthermore, *CACUL1* expression was significantly increased in tissues with adjacent hepatic inflammation (**Figure 2D**), implying a potential role in the inflammatory tumor microenvironment. In contrast, no significant difference in *CACUL1* expression was observed regarding non-alcoholic fatty liver disease status (**Figure 2E**).

### *CACUL1* serves as an independent prognostic factor for hepatocellular carcinoma patient outcomes

**Figure 3:**
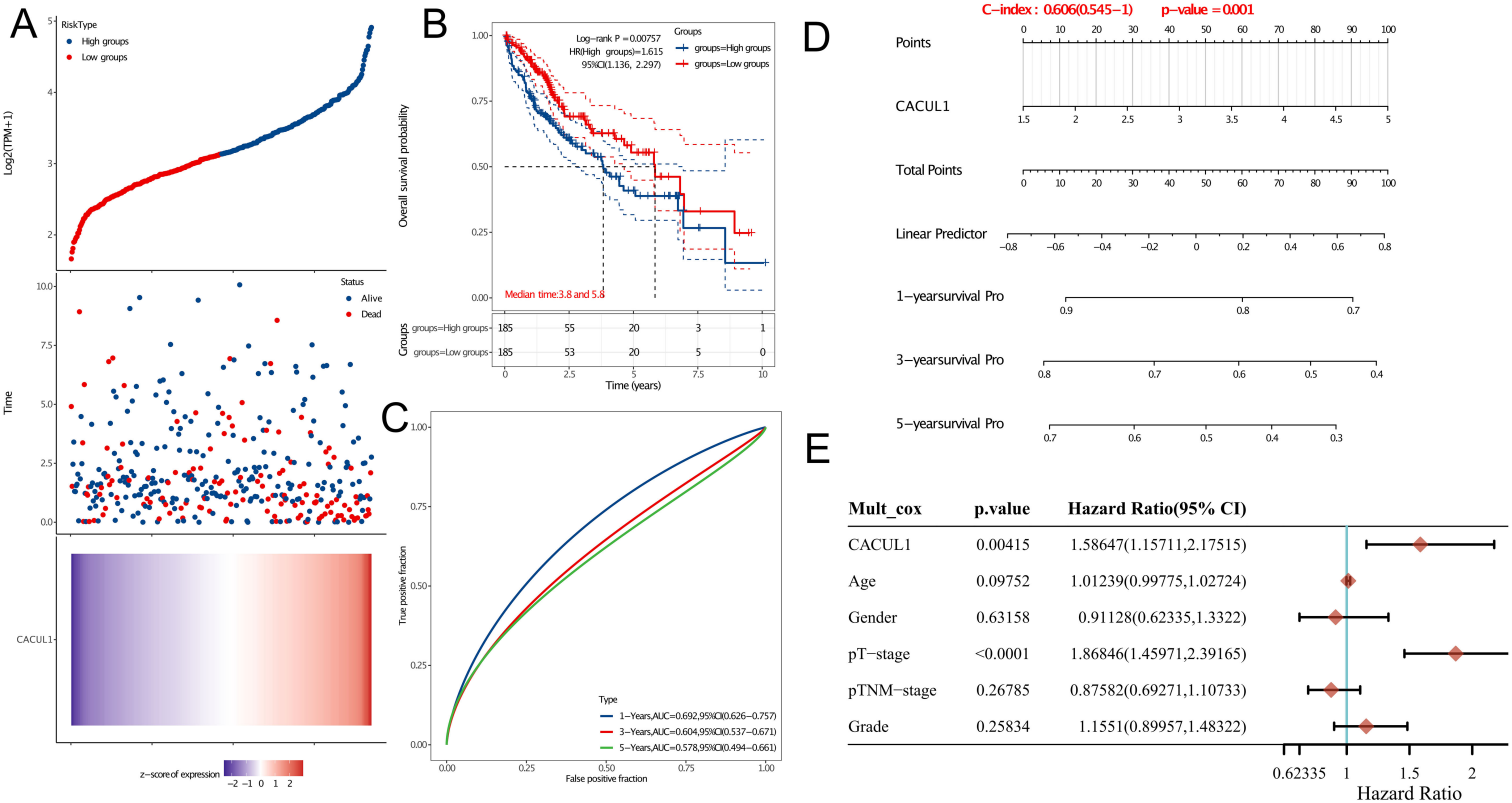
*CACUL1* serves as an independent prognostic factor for HCC. **(A)** Risk score distribution, survival status, and *CACUL1* expression heatmap for TCGA-LIHC patients stratified by *CACUL1* levels. **(B)** Kaplan–Meier curves showing poorer survival in CACUL1-high patients (median: 3.8 vs. 5.8 years; HR = 1.615, log-rank *P* = 0.00757). **(C)** Time-dependent ROC curves for 1-year (AUC = 0.692), 3-year (AUC = 0.604), and 5-year (AUC = 0.578, approaching random classification) survival prediction. **(D)** Nomogram predicting 1-, 3-, and 5-year survival incorporating *CACUL1* expression (C-index = 0.606, *P* = 0.001). **(E)** Multivariable Cox regression confirming *CACUL1* as an independent prognostic factor (HR = 1.586, *P* = 0.00415) after adjusting for clinical covariates.

To determine whether *CACUL1* serves as an independent prognostic factor in hepatocellular carcinoma, patients were stratified into high- and low-expression groups according to *CACUL1* expression (**Figure 3A**). Kaplan–Meier analysis showed that high *CACUL1* expression was associated with poorer survival (**Figure 3B**). We next assessed the prognostic performance of CACUL1 using time-dependent ROC curves. The AUC values were 0.692 (1-year), 0.604 (3-year), and 0.578 (5-year), indicating moderate short-term predictive value that declines over longer follow-up periods (**Figure 3C**). The 5-year AUC of 0.578 approaches the threshold of random classification (AUC = 0.5), suggesting limited long-term prognostic utility for CACUL1 as a standalone biomarker. The nomogram C-index of 0.606 further supports this modest predictive performance (**Figure 3D**). These findings indicate that while CACUL1 contributes to prognostic stratification, its predictive accuracy is comparable to or lower than established clinical staging systems such as BCLC (C-index ~0.6-0.7) and CLIP score (C-index ~0.6-0.65), highlighting the need for integration with conventional clinicopathological variables. Finally, in a multivariable Cox proportional hazards model adjusting for conventional clinicopathological variables, *CACUL1* remained independently associated with prognosis in hepatocellular carcinoma (**Figure 3E**).

### Elevated *CACUL1* expression is associated with altered immune checkpoint profiles and macrophage infiltration in hepatocellular carcinoma

**Figure 4:**
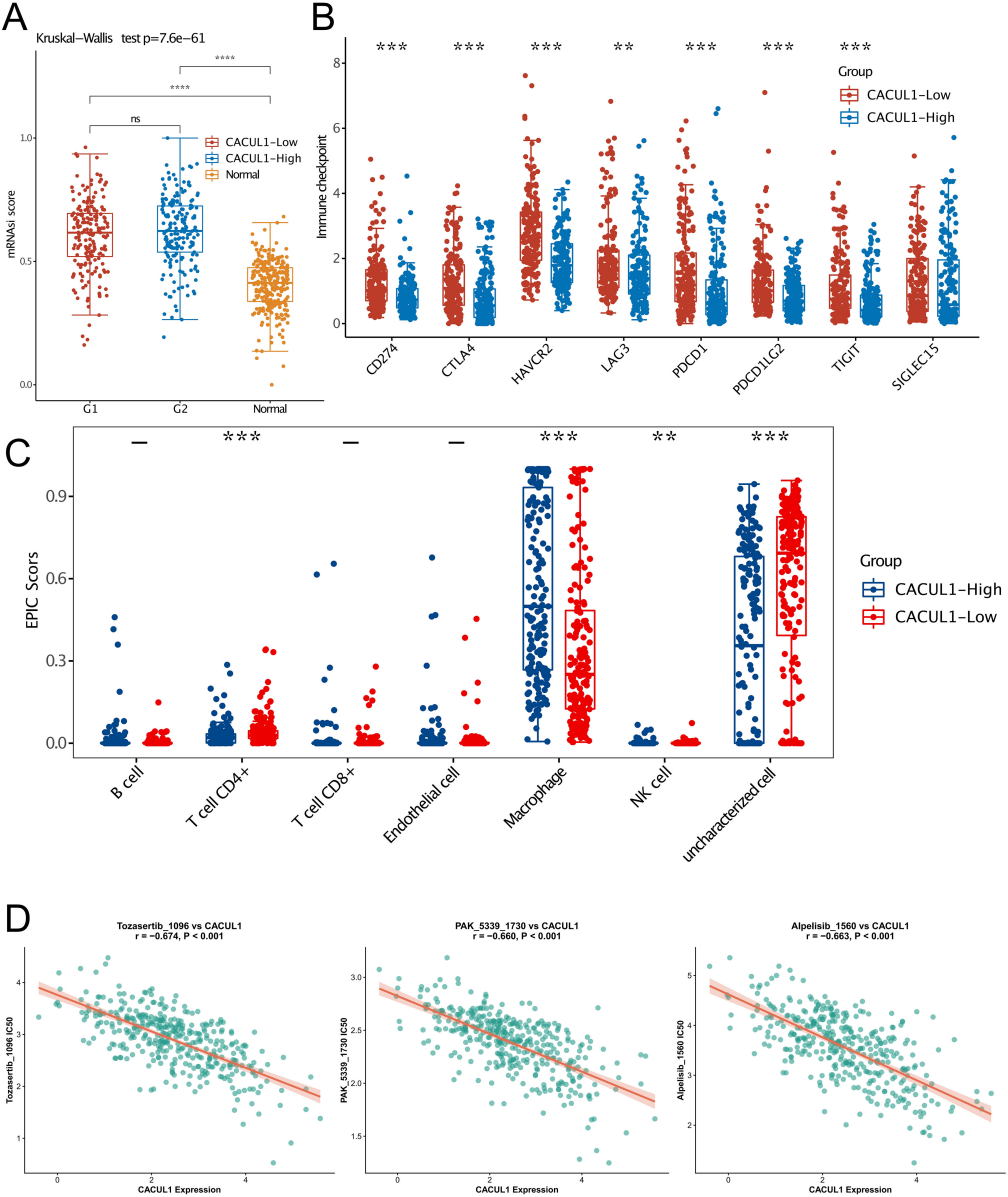
*CACUL1* expression is associated with altered immune checkpoint profiles and macrophage infiltration in HCC. **(A)** Tumor stemness (mRNAsi) scores among CACUL1-Low, CACUL1-High, and Normal groups across histological grades. **(B)** Immune checkpoint expression (CD274, CTLA4, HAVCR2, LAG3, PDCD1, PDCD1LG2, TIGIT, SIGLEC15) between CACUL1-High and CACUL1-Low groups. CACUL1-High tumors showed reduced checkpoint expression (**P < 0.01; ***P < 0.001). **(C)** EPIC immune cell infiltration analysis showing elevated macrophage scores in CACUL1-High tumors (**P < 0.01; ***P < 0.001). **(D)** Correlation between *CACUL1* expression and predicted IC50 values for Tozasertib (r = −0.674), PAK_5339_1730 (r = −0.660), and Alpelisib (r = −0.663); all *P* < 0.001.

To explore the potential implications of *CACUL1* in therapeutic responsiveness, we first assessed its correlation with tumor cell stemness. No significant association was observed between *CACUL1* expression levels and stemness indices (**Figure 4A**). Next, we investigated the relationship between *CACUL1* and key immune checkpoints. Interestingly, *CACUL1* expression was negatively correlated with the expression of CD274 (PD-L1), CTLA4, and PDCD1 (PD-1) (**Figure 4B**). This inverse correlation suggests that tumors with high *CACUL1* expression may exhibit an immune-excluded phenotype, potentially conferring resistance to immune checkpoint blockade (ICB) therapy. Beyond checkpoint markers, we analyzed immune cell infiltration profiles; elevated *CACUL1* expression was significantly associated with increased macrophage infiltration scores (**Figure 4C**), indicative of an altered immune microenvironment. Finally, we evaluated the relationship between *CACUL1* and drug sensitivity by calculating IC50 values. The results demonstrated that *CACUL1* expression levels were significantly correlated with the predicted sensitivity to several chemotherapeutic agents, including Alpelisib and Tozasertib (**Figure 4D**), highlighting its potential as a biomarker for guiding personalized treatment strategies.

### *CACUL1* promotes hepatocellular carcinoma cell proliferation associated with PI3K/AKT signaling

**Figure 5:**
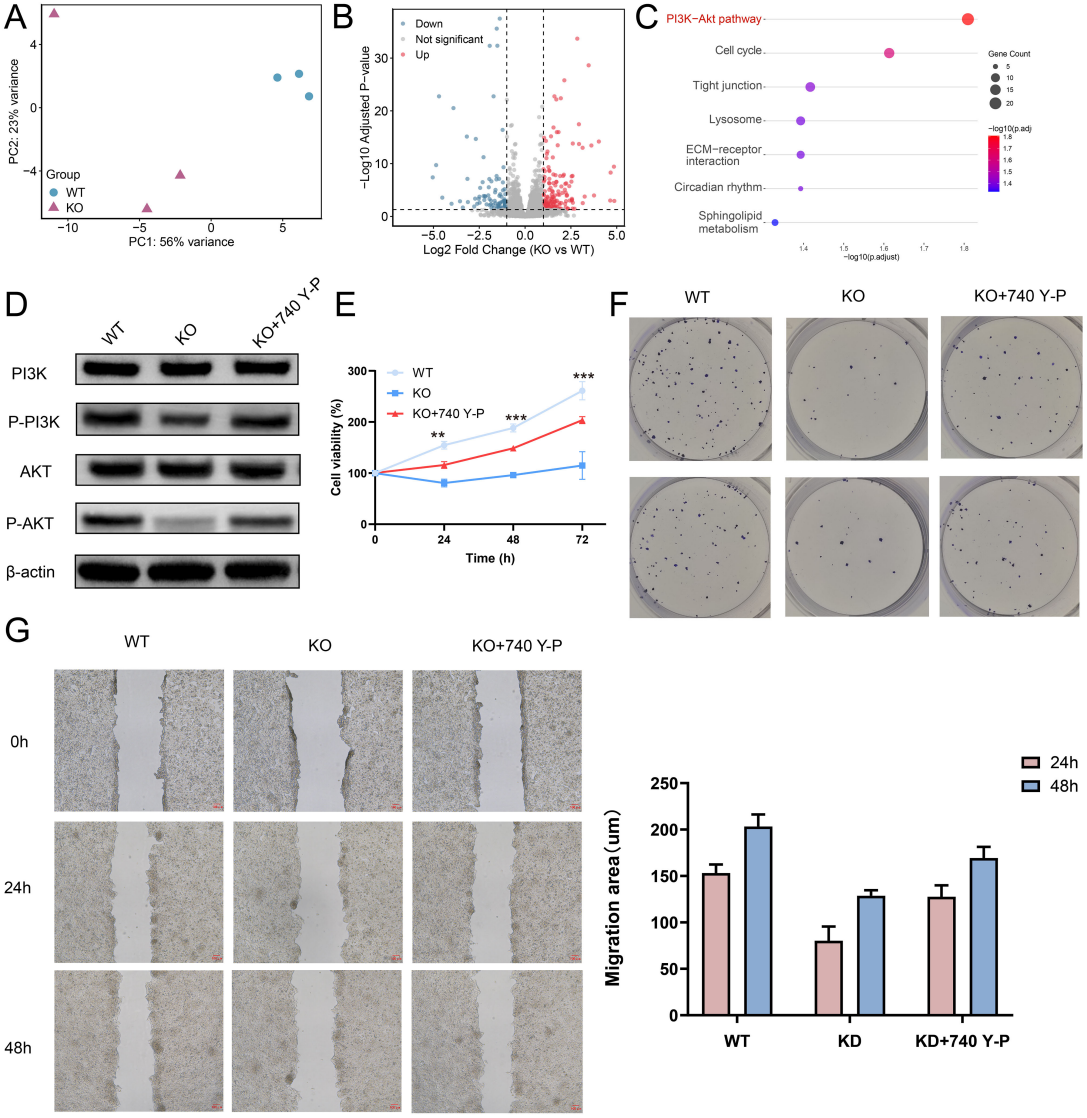
*CACUL1* promotes HCC cell proliferation in association with PI3K/AKT signaling activation. **(A)** PCA of RNA-seq data from CACUL1-KO (n = 3) and WT (n = 3) HepG2 cells (PC1: 56%, PC2: 23%). **(B)** Volcano plot of DEGs (fold change > 1.5, adjusted *P* < 0.05): 125 upregulated and 93 downregulated genes. **(C)** KEGG pathway enrichment showing PI3K-Akt signaling and cell cycle as top enriched pathways. **(D)** Western blot of PI3K, P-PI3K, AKT, and P-AKT in WT, KO and KO + 740 Y-P cells, showing decreased phosphorylation upon *CACUL1* knockdown. **(E)** CCK-8 proliferation assay showing significant growth inhibition in WT, KO and KO + 740 Y-P cells (*P* < 0.001). **(F)** Colony formation assay demonstrating reduced clonogenicity in WT, KO and KO + 740 Y-P cells. **(G)** Wound healing assay showing decreased migration in WT, KO and KO + 740 Y-P cells at 24h (*P < 0.05) and 48h (**P < 0.01).

To functionally characterize *CACUL1* in hepatocellular carcinoma, we generated CACUL1-knockout (KO) HepG2 cell lines using CRISPR/Cas9 (**Supplementary Figure 1**). Whole transcriptome profiling of CACUL1-KO cells (n=3) versus wild-type controls (n=3) revealed distinct clustering by principal component analysis (**Figure 5A**). Differential expression analysis identified 125 significantly upregulated and 93 significantly downregulated genes (fold change > 1.5; adjusted p < 0.05) (**Figure 5B**). Gene ontology and pathway enrichment analyses of the dysregulated transcriptome showed significant enrichment in PI3K-Akt signaling and cell cycle checkpoint pathways (**Figure 5C**). To validate these transcriptomic findings and the functional dependence on this pathway, we conducted rescue experiments using the specific PI3K activator 740 Y-P. Western blot analysis demonstrated that CACUL1 knockout markedly reduced the phosphorylation levels of PI3K (P-PI3K) and AKT (P-AKT) in both HepG2 and Huh7 cells, which were successfully restored back to baseline levels upon 740 Y-P treatment (**Figure 5D**; **Supplementary Figure 2A**). Consistent with these molecular alterations, functional assays, including CCK-8 and colony formation, revealed that CACUL1 knockout significantly inhibited cell proliferation and clonogenic ability, whereas 740 Y-P administration effectively rescued the growth capacity in both cell lines (**Figures 5E, F**; **Supplementary Figures 2B, C**). Furthermore, wound healing experiments demonstrated that the absence of CACUL1 significantly impaired cell migratory capacity, an effect that was also successfully reversed by the PI3K activator (**Figure 5G**; **Supplementary Figure 2D**). Collectively, these findings establish that CACUL1 positively regulates hepatocellular carcinoma cell proliferation and migration primarily through the activation of the PI3K/AKT signaling pathway.

### *CACUL1* suppresses Notch1 signaling to promote M2 macrophage polarization in the tumor microenvironment

**Figure 6:**
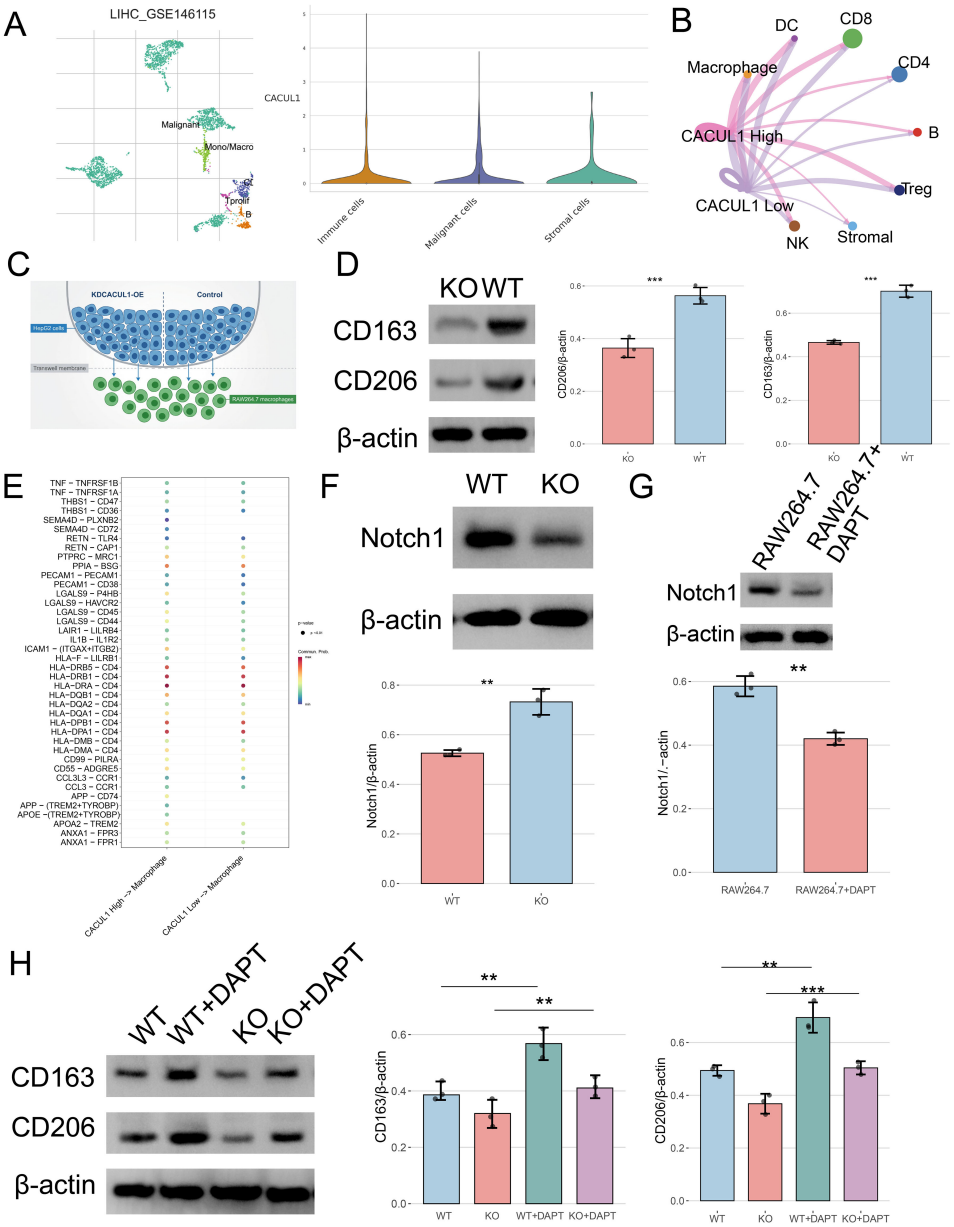
*CACUL1* suppresses Notch1 signaling to promote M2 macrophage polarization in the tumor microenvironment. **(A)** scRNA-seq analysis of the GSE146115 HCC dataset. Left: t-SNE plot showing major cell populations (Malignant, Mono/Macro, T, Prolif, B cells). Right: violin plot confirming *CACUL1* predominant expression in malignant hepatocytes. **(B)** CellChat network diagram showing differential cell-cell communication between CACUL1-high and CACUL1-low tumor cells and macrophages. Line thickness indicates communication probability; color intensity indicates interaction strength. **(C)** Schematic of the Transwell co-culture system: HepG2 cells (WT, KO, or CACUL1-OE) in the upper chamber and RAW264.7 macrophages in the lower chamber. **(D)** Western blot and quantification of M2 markers CD163 and CD206 in RAW264.7 macrophages co-cultured with WT or CACUL1-KO HepG2 cells. *CACUL1* knockout significantly attenuated M2 marker expression (***P < 0.001). **(E)** Dot plot of differential ligand-receptor interactions. Dot size represents communication probability; color represents interaction strength (red: enhanced in CACUL1-high, blue: enhanced in CACUL1-low). **(F)** Western blot and quantification showing elevated Notch1 expression in macrophages co-cultured with CACUL1-KO versus WT HepG2 cells (**P < 0.01). **(G)** Western blot confirming effective pharmacological inhibition of Notch1 in RAW264.7 cells by the γ-secretase inhibitor DAPT (**P < 0.01). **(H)** Western blot and quantification of CD163 and CD206 across WT, WT+DAPT, KO, and KO+DAPT co-culture conditions. DAPT treatment rescued M2 polarization even in CACUL1-KO cells, confirming that *CACUL1* promotes M2 macrophage polarization through Notch1 pathway suppression (**P < 0.01; ***P < 0.001).

Given the observed positive correlation between *CACUL1* expression and macrophage infiltration, we hypothesized that *CACUL1* may modulate macrophage polarization within the tumor microenvironment. To address this, we first interrogated the GSE146115 single-cell RNA-sequencing dataset and confirmed that *CACUL1* is predominantly expressed in malignant hepatocytes (**Figure 6A**). Cell-cell interaction analysis using CellChat analysis comparing CACUL1 expression-defined tumor cell subgroups (high vs. low) revealed differential putative crosstalk with macrophages (**Figure 6B**), prompting functional validation in co-culture systems. HepG2 cells (wild-type or CACUL1-KO) were co-cultured with RAW264.7 macrophages in a Transwell system (**Figure 6C**), and M2 polarization markers were assessed by Western blot and flow cytometry. Co-culture with wild-type HepG2 cells significantly upregulated the M2 markers CD163 and CD206 in macrophages (p < 0.01), whereas *CACUL1* knockout attenuated this effect (**Figure 6D**). To identify candidate signaling pathways mediating this crosstalk, we performed differential ligand-receptor interaction analysis between CACUL1-high and CACUL1-low tumor cells, identifying 24 pathways with significant alterations (**Figure 6E**). Among differentially expressed ligands in our bulk RNA-seq data, TNF was notably downregulated upon CACUL1 knockout. Of the 24 differentially active pathways identified by CellChat, TNF and Notch signaling were prioritized for subsequent validation because both pathways showed consistent directionality across multiple ligand-receptor pairs, TNF was concordantly differentially expressed in our bulk RNA-seq data, and Notch1 has established roles in macrophage polarization, making it a mechanistically plausible candidate for functional testing. Given that TNF can modulate Notch signaling and Notch1 has been implicated in macrophage function, we examined Notch1 expression in co-cultured macrophages. As expected, *CACUL1* knockout resulted in elevated Notch1 signaling in macrophages (**Figure 6F**), suggesting that *CACUL1* may suppress Notch1 activation. To test whether Notch1 functionally mediates the suppression of M2 polarization, we treated the co-culture system with the γ-secretase inhibitor DAPT to block Notch signaling (**Figure 6G**). Pharmacological inhibition of Notch1 significantly increased the M2 markers CD163 and CD206 (p < 0.01) (**Figure 6H**), even in CACUL1-deficient cells, indicating that *CACUL1* promotes M2 macrophage polarization through inhibition of the Notch1 signaling pathway. Conversely, to determine whether Notch1 inhibition is an indispensable step, we performed a Notch1 activation rescue experiment using the Notch ligand DLL4-Fc. Immunoblot analysis confirmed that DLL4-Fc treatment successfully activated Notch1 signaling, as evidenced by increased Cleaved Notch1 (NICD) in RAW264.7 cells (**Supplementary Figure 3A**). Notably, the M2 polarization induced by WT HepG2 co-culture was remarkably reversed by the forced activation of Notch1 via DLL4-Fc (**Supplementary Figure 3B**), confirming the necessity of Notch1 suppression.

Furthermore, we sought to definitively establish whether TNF-α acts as the crucial intermediate mediator linking CACUL1 to Notch1 suppression. Immunoblotting confirmed that WT HepG2 cells expressed higher levels of TNF-α compared to CACUL1-KO cells (**Supplementary Figure 3C**). In the co-culture system, neutralization of TNF-α using Infliximab successfully restored Notch1 expression and abrogated the WT-induced M2 macrophage polarization (**Supplementary Figure 3D**). Reciprocally, supplementing the CACUL1-KO co-culture system with exogenous recombinant TNF-α re-suppressed Notch1 and rescued the M2 polarization phenotype (**Supplementary Figure 3E**). Collectively, these findings provide compelling causal evidence that CACUL1 drives M2 macrophage polarization specifically through the TNF-α/Notch1 signaling cascade.

### *CACUL1* knockdown suppresses tumor growth and M2 macrophage polarization in hepatocellular carcinoma

**Figure 7:**
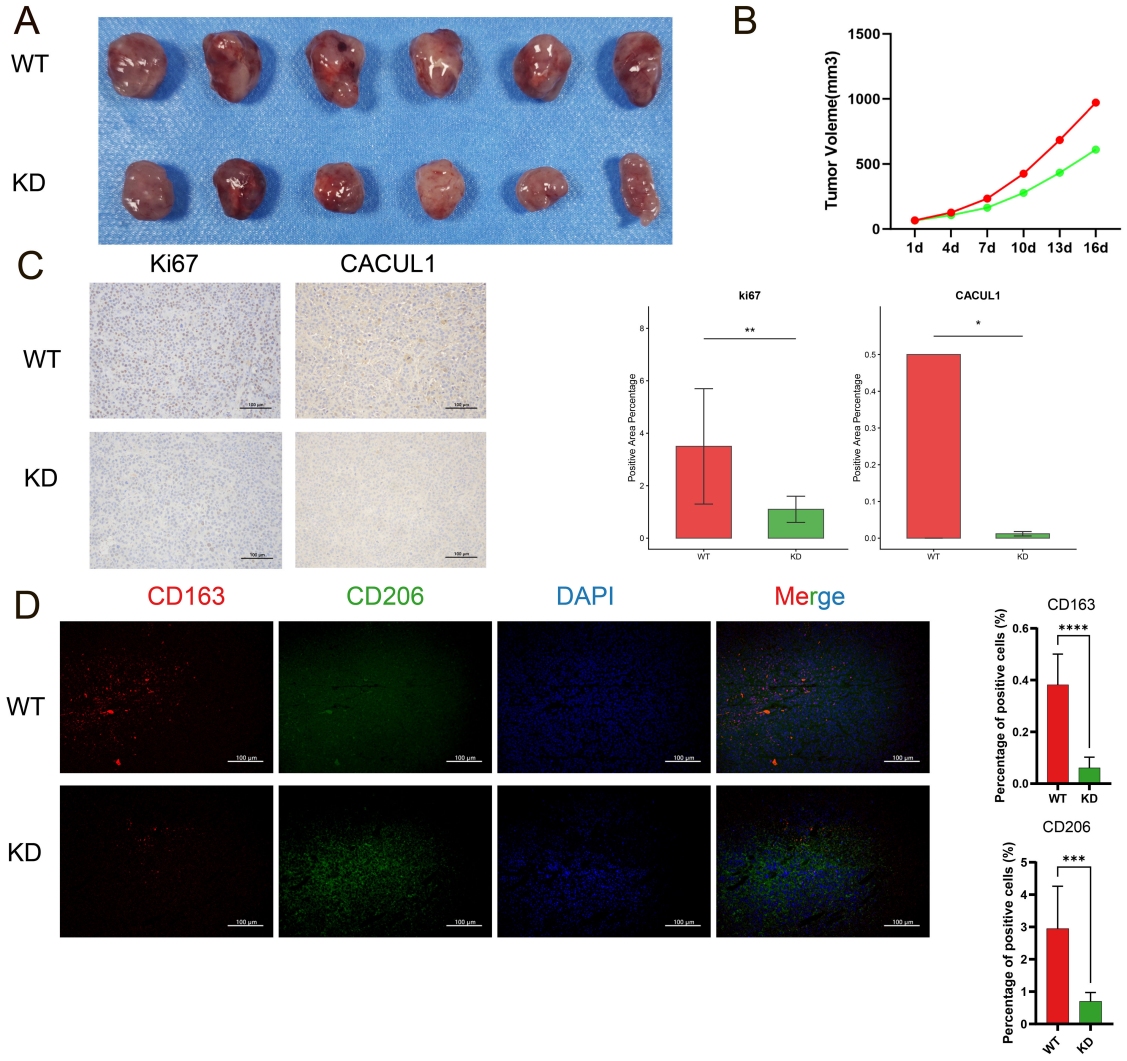
*CACUL1* knockdown suppresses tumor growth and M2 macrophage polarization *in vivo*. **(A)** Representative xenograft tumors from WT and CACUL1-KD groups (n = 6 per group). **(B)** Tumor growth curves over 16 days showing reduced tumor volume in KD mice. **(C)** IHC staining showing higher Ki67 expression in WT versus KD tumors (**P < 0.01) and confirming *CACUL1* knockdown efficiency (*P < 0.05). **(D)** Representative immunofluorescence of CD163 (red) and CD206 (green) with DAPI counterstaining, qualitatively showing M2 macrophage marker expression in WT and KD tumors. Quantitative analysis was not performed. *** indicates P < 0.001; **** indicates P < 0.0001.

To investigate the role of *CACUL1* in hepatocellular carcinoma, we performed an *in vivo* xenograft experiment in mice to assess its effects on tumor growth and M2 macrophage polarization. Tumors in the *CACUL1* knockdown (KD) group were smaller than those in the wild-type (WT) group, and their growth rate was also reduced (**Figures 7A, B**). Consistently, Ki67 immunohistochemical staining demonstrated higher Ki67 expression in the WT group than in the KD group (**Figure 7C**). Furthermore, qualitative immunofluorescence staining revealed visibly enhanced CD163 and CD206 fluorescence signals in tumors from WT mice compared with those from KD mice (**Figure 7D**). These images are representative of the observed staining patterns and are not intended for quantitative comparison.

## Discussion

**Figure 8:**
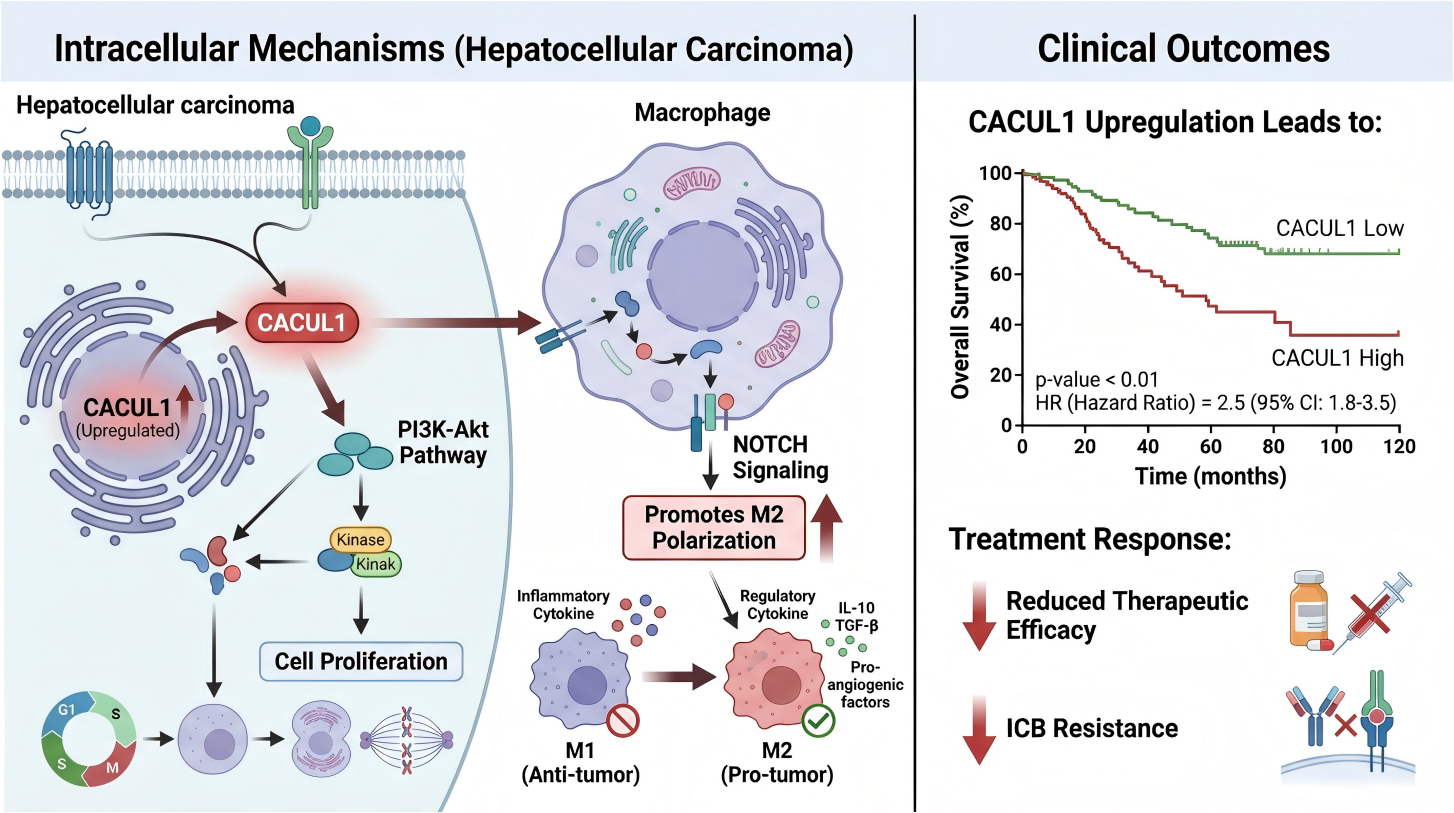
Graphical abstract illustrating the dual oncogenic mechanisms of CACUL1 in hepatocellular carcinoma. Left panel: In hepatocellular carcinoma cells, upregulated CACUL1 activates the PI3K-Akt signaling pathway, enhancing kinase activity and promoting tumor cell proliferation, thereby promoting tumor cell proliferation. Middle panel: CACUL1 suppresses Notch signaling in tumor-associated macrophages through paracrine crosstalk, driving M1-to-M2 macrophage polarization, as evidenced by upregulated M2 markers CD163 and CD206 (**Figures 6D**, **7D**), which fosters an immunosuppressive tumor microenvironment. Right panel: Clinically, CACUL1 upregulation is associated with significantly reduced overall survival (HR = 2.5, 95% CI: 1.8–3.5, P < 0.01), potential association with reduced responsiveness to immune checkpoint blockade, which requires validation in treatment-annotated cohorts.

This study reveals the molecular mechanism of CACUL1 as a dual-axis oncogenic driver in hepatocellular carcinoma (HCC). In tumor cells, CACUL1 upregulation enhances downstream kinase activity by activating the PI3K-Akt signaling pathway, thereby promoting tumor cell proliferation. At the same time, CACUL1 modulates macrophage polarization in the tumor microenvironment through paracrine crosstalk. Our co-culture experiments showed that CACUL1 expression in HCC cells was associated with reduced Notch1 signaling in macrophages and increased expression of the M2 markers CD163 and CD206, whereas CACUL1 knockdown attenuated this phenotype. These findings suggest that CACUL1 may contribute to macrophage-mediated immune suppression, although the specific soluble mediators and downstream cytokine changes remain to be further defined. At the clinical outcome level, high expression of CACUL1 is significantly associated with a shorter overall survival in HCC patients (HR = 2.5, P < 0.01) and is closely related to reduced efficacy of immunotherapy and resistance to immune checkpoint blockade (ICB). In summary, CACUL1 drives the progression of HCC by coordinating the enhancement of both cell proliferation and immune escape mechanisms, and is a potential prognostic marker and therapeutic target (**Figure 8**).

Our findings that *CACUL1* is upregulated in hepatocellular carcinoma and promotes tumor cell proliferation are consistent with its established roles in other malignancies. *CACUL1* has been functionally implicated in enhancing cell cycle progression and tumor cell survival, with documented overexpression in gastric, colorectal, and lung cancers, as well as glioma, where it drives tumor growth, chemoresistance, and stress adaptation. In gastric cancer, for example, upregulation of circ−ERBB2 relieves miR−503–mediated repression of CACUL1, thereby facilitating proliferation, invasion, and metastasis and predicting unfavorable prognosis, while Helicobacter pylori can activate AP−1 to induce *CACUL1* expression and further drive invasive behavior (9, 10). Similarly, publicly available clinical resources such as the Human Protein Atlas indicate that high *CACUL1* expression is an adverse prognostic marker in KIRC, hepatocellular carcinoma, and ovarian serous carcinoma. Our pan−cancer analysis both corroborated and extended these observations (11). We identified significant differential expression of *CACUL1* specifically in liver hepatocellular carcinoma and gastric cancer, yet found that *CACUL1* expression was associated with patient outcome across 16 distinct tumor types. This discordance—limited transcriptional dysregulation but widespread prognostic impact—suggests that *CACUL1* primarily influences tumor progression and aggressiveness rather than tumor initiation. Such context−dependent functionality is exemplified by our observation that *CACUL1* exerts divergent prognostic effects in KIRC and KIRP. Although these renal cancer subtypes share a subset of key microRNAs, they differ markedly in miRNA–target interaction networks, genomic landscapes, epigenetic states, and tumor microenvironments, potentially explaining why *CACUL1* integrates into pro−tumorigenic signaling in one subtype but not the other. This pattern is consistent with reports that individual genes can display opposite prognostic implications in KIRC versus KIRP; for example, high BRPF3 expression correlates with improved survival in KIRC but worse outcomes in KIRP, and large−scale pan−cancer analyses have shown that survival−related genes often exhibit highly cancer type–specific associations with prognosis (12). Collectively, these findings underscore the importance of context−dependent functional validation and suggest that therapeutic targeting of *CACUL1* should be tailored to specific tumor types and molecular subtypes.

The distinct prognostic effects of *CACUL1* across multiple malignancies may be mechanistically linked to its influence on the tumor immune microenvironment, particularly through altered immune checkpoint expression. In our pan-cancer analysis, *CACUL1* expression was negatively correlated with the expression of multiple co-inhibitory immune checkpoint molecules, including PD-L1, PD-1, and LAG-3, in hepatocellular carcinoma (**Figure 4B**). This inverse correlation indicates that *CACUL1*-high tumors exhibit reduced immune checkpoint expression, which is inconsistent with a conventional “hot” inflamed phenotype characterized by high checkpoint levels and abundant T-cell infiltration. Instead, this pattern suggests an immune-excluded phenotype, wherein physical or functional barriers prevent T-cell penetration into the tumor parenchyma despite the presence of peripheral immune cells. In this context, reduced checkpoint expression may reflect insufficient T-cell priming and activation rather than active immune evasion through checkpoint upregulation. *CACUL1* enhances tumor cell proliferation and survival by promoting CDK2 kinase activity and PI3K/AKT signaling. While emerging evidence suggests that cell cycle-active tumor cells can upregulate immune checkpoint molecules through interferon-gamma-mediated mechanisms in inflamed tumors (13, 14), our data reveal a distinct pattern in *CACUL1*-high HCC: these tumors show reduced checkpoint expression alongside increased macrophage infiltration (**Figures 4B, C**). This discordance suggests that *CACUL1*-driven immune evasion operates through macrophage-mediated mechanisms rather than conventional checkpoint-dependent pathways. M2-polarized tumor-associated macrophages (TAMs) can physically exclude T cells from tumor nests through extracellular matrix remodeling and establish immunosuppressive niches via cytokine secretion (IL-10, TGF-β), thereby creating a “cold” microenvironment with low checkpoint expression and poor T-cell infiltration (15–17). In hepatocellular carcinoma, this immune-excluded phenotype is associated with aggressive disease and resistance to immune checkpoint blockade (ICB) therapy, as the absence of pre-existing T-cell immunity limits the efficacy of checkpoint inhibitors (18, 19). Thus, *CACUL1-*high tumors may represent a subset of HCC patients who would not benefit from ICB monotherapy and may require combination strategies targeting macrophage polarization or T-cell recruitment (20, 21). While this study focuses on CACUL1-mediated macrophage polarization, the HCC immune microenvironment comprises diverse cellular players including neutrophils, monocytes, dendritic cells, NK cells, and T cells. The impact of CACUL1 on these additional immune cell types was not investigated and represents an important direction for future research. Collectively, these findings suggest that CACUL1 drives HCC progression through a dual mechanism encompassing both enhanced tumor cell proliferation and macrophage-mediated immune suppression. Unlike conventional immune evasion through checkpoint upregulation, CACUL1 promotes an immune-excluded microenvironment characterized by M2-dominant macrophage infiltration and reduced T-cell presence, positioning it as a distinct therapeutic target for reprogramming the tumor immune landscape. CACUL1 differs from single-pathway drivers such as MYC or PD-L1 by simultaneously promoting proliferation and immune evasion, and from canonical cullins through its non-canonical CDK2-associated mechanism.

While our analysis demonstrates that CACUL1 expression correlates with advanced TNM stage and poor survival, prospective validation in independent cohorts and evaluation of its predictive value for specific therapeutic responses are beyond the scope of this study. Future clinical trials should assess whether CACUL1 stratification can guide treatment decisions, such as selecting patients for PI3K/AKT inhibitors or macrophage-targeted combination therapies.

To comprehensively elucidate the functional role of *CACUL1* in hepatocellular carcinoma, we performed transcriptome sequencing of CACUL1-knockout cells and conducted pathway enrichment analysis, which identified PI3K/AKT and cell cycle signaling as primary targets of *CACUL1* regulation. Subsequent *in vitro* and *in vivo* studies confirmed that *CACUL1* knockdown significantly inhibited hepatocellular carcinoma cell proliferation and migration but did not substantially affect invasiveness in transwell invasion assays. This selective impact on migration without concomitant modulation of invasion warrants mechanistic consideration. Migration and invasion, though often conflated, represent biologically distinct processes with divergent molecular requirements. Cell migration is primarily governed by cytoskeletal reorganization and integrin-mediated cell-matrix adhesion. The PI3K/AKT pathway, which we found to be downstream of CACUL1, is well established to enhance cell motility through regulation of cytoskeletal dynamics and focal adhesion turnover. In contrast, cell invasion typically requires proteolytic degradation of basement membrane components via matrix metalloproteinases (MMPs), a process predominantly controlled by epithelial-mesenchymal transition (EMT) programs involving TGF-β, Wnt, and Notch signaling (22, 23). Our transcriptomic data did not reveal significant enrichment of EMT-related gene signatures upon *CACUL1* knockout (data not shown), suggesting that *CACUL1* does not directly regulate EMT transcriptional programs (24). The dissociation between CACUL1-driven proliferation/migration and its lack of effect on invasion is also consistent with the “go or grow” model, which posits that cancer cell proliferation and invasion represent largely mutually exclusive cellular states. According to this framework, cells engaged in PI3K/AKT-driven cell cycle progression may allocate metabolic resources and signaling pathways toward proliferation at the expense of energy-intensive invasive programs (25, 26). Notably, several studies in hepatocellular carcinoma have reported similar phenotypic dissociations, wherein oncogenes promoting proliferation do not necessarily confer invasive capacity unless accompanied by EMT activation or stromal remodeling. For example, mTORC1 hyperactivation enhances HCC cell proliferation but can paradoxically suppress invasion through negative feedback on PI3K signaling. Collectively, these findings indicate that *CACUL1* promotes hepatocellular carcinoma progression primarily through enhanced proliferative and migratory capacity via PI3K/AKT signaling, but does not independently activate the EMT program or proteolytic machinery necessary for basement membrane penetration (14, 27, 28). This suggests that CACUL1-driven tumors may require additional microenvironmental cues—such as TGF-β from tumor-associated fibroblasts or hypoxia-induced EMT—to acquire full metastatic potential. Future studies integrating *CACUL1* expression with EMT markers and stromal signatures in clinical HCC specimens may clarify whether CACUL1-high tumors exhibit distinct metastatic patterns or therapeutic vulnerabilities.

Beyond its cell-intrinsic effects on tumor cell proliferation and survival, *CACUL1* exerts profound immunomodulatory functions within the tumor microenvironment, particularly through reprogramming of macrophage polarization. Our transcriptomic and single-cell sequencing analyses revealed that *CACUL1* expression is associated with an altered immune landscape characterized by enhanced infiltration of tumor-associated macrophages (TAMs) with an M2 immunosuppressive phenotype. Functional validation in co-culture experiments confirmed that CACUL1-expressing HCC cells actively promote M2 macrophage polarization through suppression of Notch1 signaling. These findings align with the established role of Notch signaling in macrophage polarization. Canonical Notch activation—mediated by engagement of Notch receptors with Delta-like ligands (DLL4) and Jagged proteins—promotes M1 polarization and anti-tumor macrophage function through induction of interferon regulatory factor 8 and transcription of M1-associated genes (IL-12, iNOS) (29, 30). Conversely, suppression or blockade of Notch signaling permits skewing toward a pro-tumor M2 phenotype. Our observation that *CACUL1* inhibits Notch1 in macrophages is therefore mechanistically consistent with the observed increase in M2 markers (CD163, CD206). Additionally, Notch signaling regulates the expression of signal regulatory protein alpha (SIRPα), which inhibits macrophage phagocytosis of CD47-expressing tumor cells; thus, CACUL1-mediated Notch suppression may also impair macrophage-mediated tumor clearance. In general, M2-polarized TAMs have been reported to support tumor progression through secretion of immunosuppressive cytokines, growth factors, and matrix-remodeling enzymes. However, in the present study, we did not directly measure these cytokines or angiogenic mediators. Therefore, our conclusion is limited to the observation that CACUL1 promotes an M2-like macrophage phenotype, as indicated by CD163 and CD206 upregulation, together with altered Notch1 signaling (15–17). Third, they establish metabolically supportive niches through enhanced oxidative phosphorylation and fatty acid catabolism that accommodate tumor metabolic demands. Notably, CACUL1-driven tumor cell proliferation via the PI3K/AKT pathway may further amplify this immunosuppressive state (31). Rapidly proliferating tumor cells exhibit enhanced glycolysis and lactate production, which can be sensed by tumor-infiltrating macrophages via GPR132 to reinforce M2 polarization, potentially establishing a feed-forward loop wherein CACUL1-driven tumor progression fosters an immunologically “cold” microenvironment (32). Collectively, these observations indicate that *CACUL1* functions as a dual-purpose oncogenic driver: it enhances intrinsic tumor cell malignancy through CDK2-associated proliferative signaling and PI3K/AKT activation, while simultaneously remodeling the immune microenvironment toward an immunosuppressive state dominated by pro-tumor M2 macrophages via Notch1 suppression (33). This dual mechanism suggests that therapeutic targeting of CACUL1—either through direct inhibition or by disrupting its downstream effectors—may yield synergistic benefits by both impeding tumor cell proliferation and reprogramming the immunosuppressive microenvironment. Future studies should evaluate whether *CACUL1* inhibition can restore Notch-dependent M1 polarization and enhance the efficacy of immune checkpoint blockade or adoptive cell therapy in hepatocellular carcinoma.

The modest C-index (0.606) and declining AUC over time suggest that CACUL1 alone has limited long-term prognostic value and may serve best as a complementary biomarker integrated with conventional staging. The complexity of the hepatocellular carcinoma microenvironment presents both challenges and opportunities for therapeutic intervention. Recent studies have highlighted the intricate interplay between tumor cells, immune cells, and stromal components in shaping HCC progression and treatment response (34–36). For example, Donne et al. (34) provided a comprehensive overview of the HCC immune microenvironment, emphasizing the diverse cellular players including neutrophils, monocytes, macrophages, dendritic cells, NK cells, and T cells, each contributing to distinct immunotherapy modalities. The specific impact of CACUL1 on these additional immune cell types was beyond the scope of this study and warrants future investigation. Han et al. (35)further elucidated RNA-binding protein-mediated immune evasion mechanisms through PD-L1 regulation, revealing alternative pathways for immune checkpoint modulation independent of conventional IFN-γ signaling. These findings underscore that HCC immune evasion operates through multiple parallel mechanisms, and CACUL1-driven M2 macrophage polarization represents one such pathway within this complex landscape. Future studies should integrate CACUL1 analysis with multi-omics profiling of the HCC microenvironment to identify patient subsets most likely to benefit from CACUL1-targeted therapies.

Several limitations of this study warrant acknowledgment and suggest directions for future investigation. First, while our transcriptomic analysis identified the PI3K/AKT pathway as a primary downstream target of CACUL1, we did not perform direct mechanistic validation through pharmacological inhibition or genetic perturbation of PI3K/AKT components. Future studies employing specific PI3K inhibitors (e.g., BYL719) or AKT inhibitors (e.g., MK-2206) in CACUL1-overexpressing cells would definitively establish causality and identify the precise nodes within this pathway that mediate CACUL1’s pro-proliferative effects. Second, the single-cell RNA sequencing analysis presents correlative rather than causal evidence. The CellChat analysis compares CACUL1-high and CACUL1-low tumor cells as defined by endogenous expression levels. These subgroups may differ in multiple transcriptomic dimensions beyond CACUL1 itself, and attributing differential cell-cell communication specifically to CACUL1 is not justified without functional perturbation. The causal role of CACUL1 in macrophage crosstalk was established through independent CRISPR knockout and pharmacological rescue experiments in our bulk co-culture system. Third, although our co-culture experiments demonstrated that *CACUL1* promotes M2 macrophage polarization via Notch1 suppression, we did not investigate whether this interaction is bidirectional—that is, whether M2-polarized macrophages can reciprocally modulate *CACUL1* expression in tumor cells through paracrine signaling. Given that M2 macrophages secrete a complex milieu of cytokines (IL-10, TGF-β) and growth factors (EGF, PDGF), some of which activate PI3K/AKT signaling, it is plausible that M2-TAMs may establish a positive feedback loop that sustains or amplifies *CACUL1* expression. Addressing this question would require reverse co-culture experiments coupled with *CACUL1* promoter activity assays or chromatin immunoprecipitation to identify transcription factors responsive to M2-derived signals. These cytokines and growth factors were not directly measured in the current study and should be investigated in future work. Additionally, PI3K/AKT pathway enrichment and altered proliferation were observed upon CACUL1 knockout, direct cell cycle analysis (e.g., flow cytometry, EdU/BrdU incorporation, or cell cycle regulatory protein assessment) was not performed in this study. Therefore, we have revised our conclusions to reflect that CACUL1 promotes proliferation in association with PI3K/AKT signaling, rather than asserting definitive cell cycle acceleration or G1/S transition. Future studies will be needed to delineate whether CACUL1’s pro-proliferative effect is mediated specifically through cell cycle modulation or additional mechanisms such as suppression of apoptosis or metabolic reprogramming.

## Conclusion

*CACUL1* functions as a dual-axis oncogenic driver in HCC through enhanced proliferation via PI3K/AKT signaling and immune evasion via Notch1-dependent macrophage polarization, positioning it as a candidate prognostic biomarker and therapeutic target.

## Data Availability

The original contributions presented in the study are included in the article/**Supplementary Material**. Further inquiries can be directed to the corresponding author.

## Supplementary material

Supplementary Figure 1 Validation of CACUL1 knockout efficiency.

Supplementary Figure 2 **(A)** Western blot of PI3K, P-PI3K, AKT, and P-AKT in WT, KO and KO + 740 Y-P Huh7 cells, showing decreased phosphorylation upon *CACUL1* knockdown. **(B)** CCK-8 proliferation assay showing significant growth inhibition in WT, KO and KO + 740 Y-P Huh7 cells (*P* < 0.001). **(C)** Colony formation assay demonstrating reduced clonogenicity in WT, KO and KO + 740 Y-P Huh7 cells. **(D)** Wound healing assay showing decreased migration in WT, KO and KO + 740 Y-P Huh7 cells at 24h, 48h and 72h. (*P<0.05, **P < 0.01; ***P < 0.001)

Supplementary Figure 3 **(A)** Western blot analysis of Notch1 in RAW264.7 macrophages cultured alone or treated with DLL4-Fc to induce Notch1 activation. **(B)** Western blot analysis and quantification of M2 markers (CD163 and CD206) in macrophages co-cultured with WT or CACUL1-KO HepG2 cells, with or without DLL4-Fc treatment (*P<0.05, **P < 0.01; ***P < 0.001, ****P<0.0001). **(C)** Western blot analysis of TNF-α protein expression in WT and CACUL1-KO HepG2 cells. **(D)** Western blot analysis of Notch1 and M2 polarization markers (CD163, CD206) in macrophages co-cultured with WT or CACUL1-KO HepG2 cells, with or without the TNF-α neutralizing antibody Infliximab. **(E)** Western blot analysis of Notch1 and M2 polarization markers (CD163, CD206) in macrophages co-cultured with WT or CACUL1-KO HepG2 cells, followed by treatment with or without exogenous recombinant TNF-α. Data are presented as mean ± SD of three independent experiments.
