# Supplementary figures and images for: CACUL1 promotes hepatocellular carcinoma progression through enhanced tumor cell proliferation and macrophage-mediated immune suppression
Source: Front Immunol. 2026 Jul 16;17:1819873. doi: 10.3389/fimmu.2026.1819873 (PMC13422430; doi:10.3389/fimmu.2026.1819873)

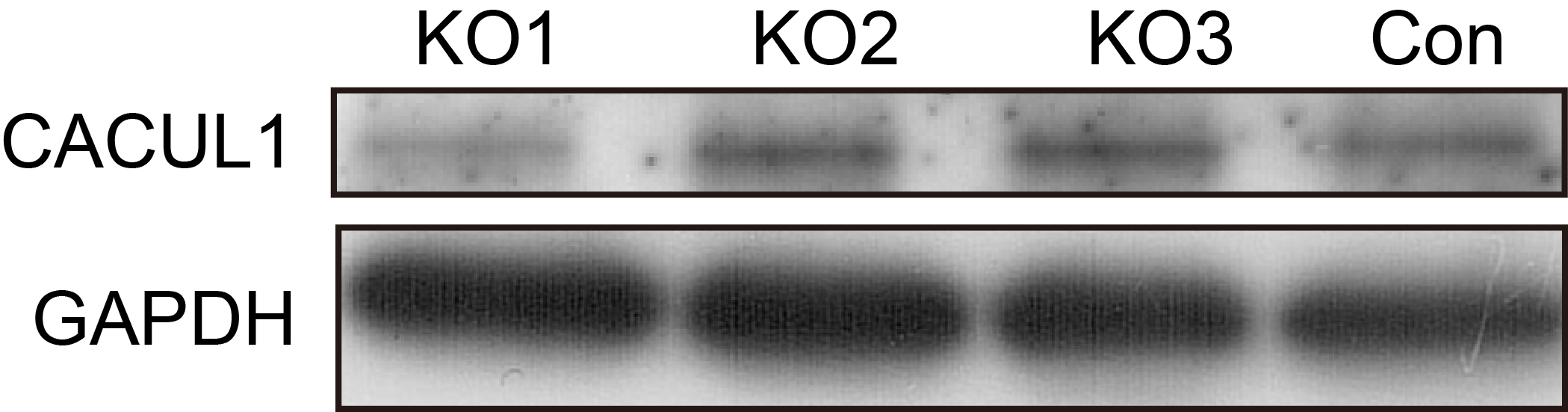

Supplement: Supplementary Figure 1 — Validation of CACUL1 knockout efficiency. [file Image1.jpeg]

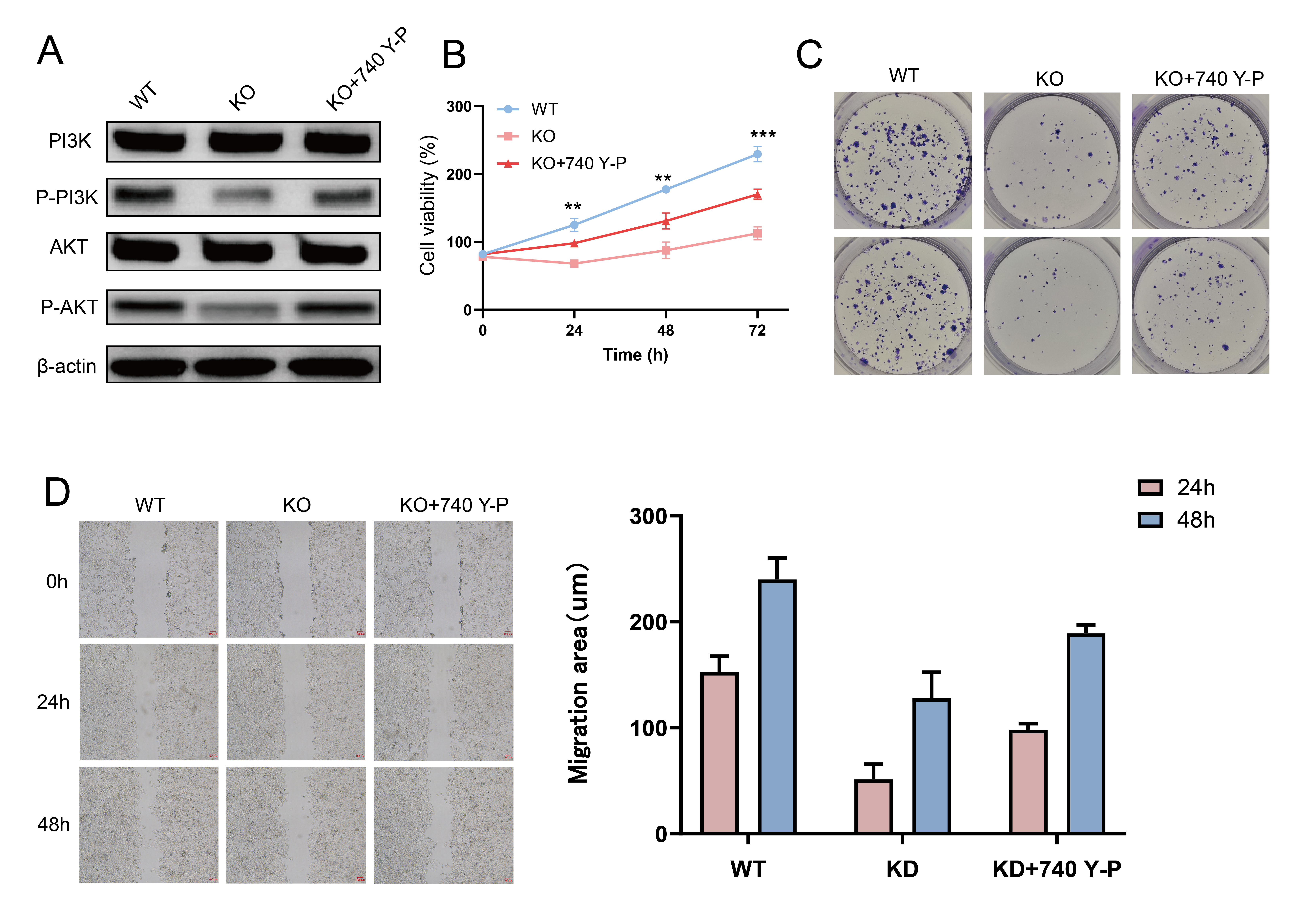

Supplement: Supplementary Figure 2 — (A) Western blot of PI3K, P-PI3K, AKT, and P-AKT in WT, KO and KO + 740 Y-P Huh7 cells, showing decreased phosphorylation upon CACUL1 knockdown. (B) CCK-8 proliferation assay showing significant growth inhibition in WT, KO and KO + 740 Y-P Huh7 cells (P < 0.001). (C) Colony formation assay demonstrating reduced clonogenicity in WT, KO and KO + 740 Y-P Huh7 cells. (D) Wound healing assay showing decreased migration in WT, KO and KO + 740 Y-P Huh7 cells at 24h, 48h and 72h. (*P<0.05, **P < 0.01; ***P < 0.001) [file Image2.jpeg]

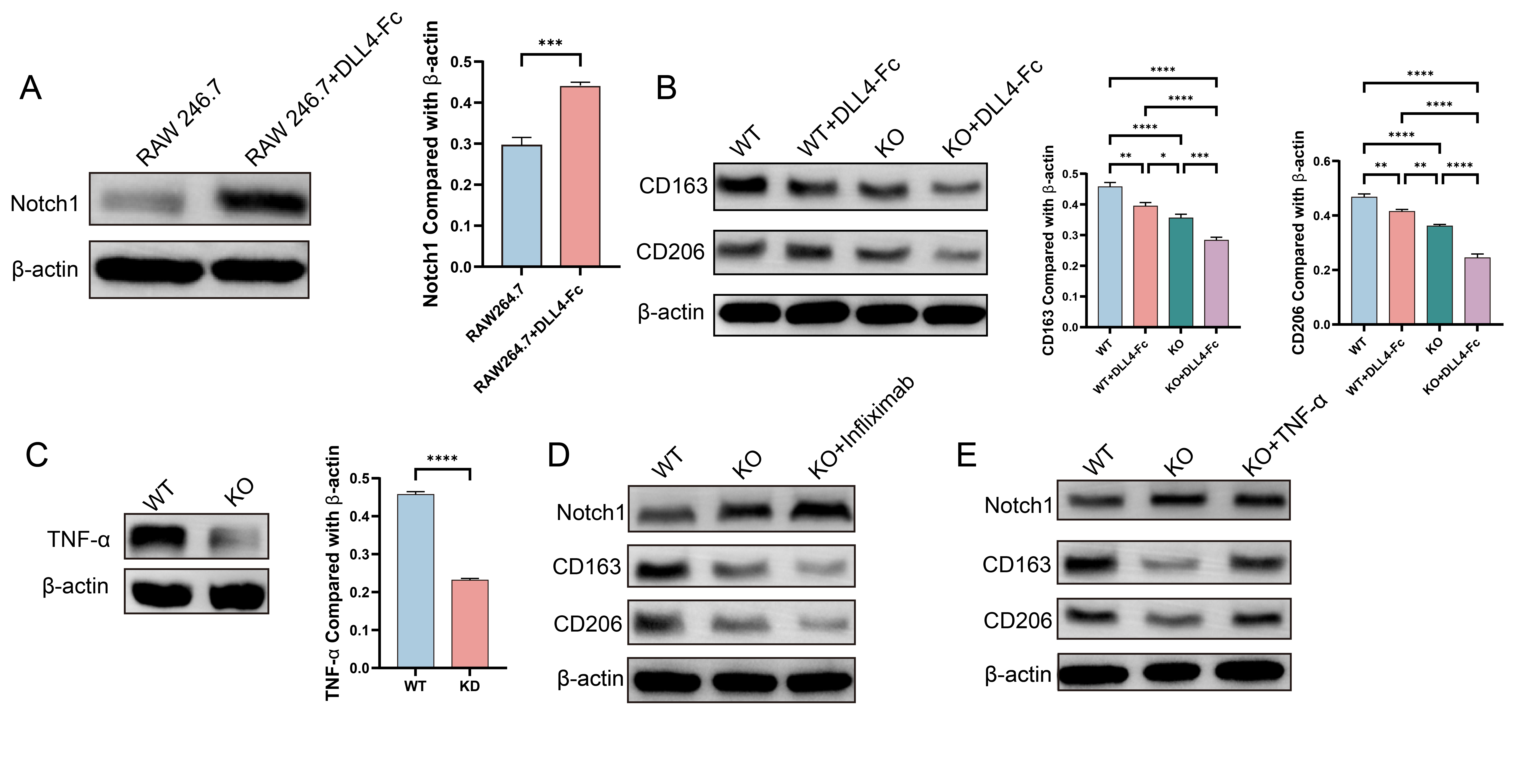

Supplement: Supplementary Figure 3 — (A) Western blot analysis of Notch1 in RAW264.7 macrophages cultured alone or treated with DLL4-Fc to induce Notch1 activation. (B) Western blot analysis and quantification of M2 markers (CD163 and CD206) in macrophages co-cultured with WT or CACUL1-KO HepG2 cells, with or without DLL4-Fc treatment (*P<0.05, **P < 0.01; ***P < 0.001, ****P<0.0001). (C) Western blot analysis of TNF-α protein expression in WT and CACUL1-KO HepG2 cells. (D) Western blot analysis of Notch1 and M2 polarization markers (CD163, CD206) in macrophages co-cultured with WT or CACUL1-KO HepG2 cells, with or without the TNF-α neutralizing antibody Infliximab. (E) Western blot analysis of Notch1 and M2 polarization markers (CD163, CD206) in macrophages co-cultured with WT or CACUL1-KO HepG2 cells, followed by treatment with or without exogenous recombinant TNF-α. Data are presented as mean ± SD of three independent experiments. [file Image3.jpeg]
